# Total synthesis and determination of the absolute configuration of a natural analgesic: crotonine

**DOI:** 10.1007/s13659-013-0080-1

**Published:** 2013-12-06

**Authors:** Yang Yang

**Affiliations:** 1 State Key Laboratory of Phytochemistry and Plant Resources in West China, Kunming Institute of Botany, Chinese Academy of Sciences, Kunming, 650201 China; 2 Graduate University of Chinese Academy of Sciences, Beijing, 100049 China

**Keywords:** total synthesis, crotonine, 2-(furan-2-yl)-5-(2,3,4-trihydroxy-butyl)-1,4-diazine, *Croton tiglium* L. (Euphoriaceae), analgesics

## Abstract

We report the first total synthesis of four possible absolute configurations and four other regional isomers of a naturally occurring alkaloid crotonine, which was isolated from *Croton tiglium* L. (Euphoriaceae) without elucidation of its absolute configuration. The concise five-step route with a chirally poor and regioselective strategy starting from monosaccharides was established, and the absolute structure of the natural crotonine was determined by comparison of the NMR spectra and optical rotations of the synthetic products.

**Electronic Supplementary Material:**

Supplementary material is available for this article at 10.1007/s13659-013-0080-1 and is accessible for authorized users.

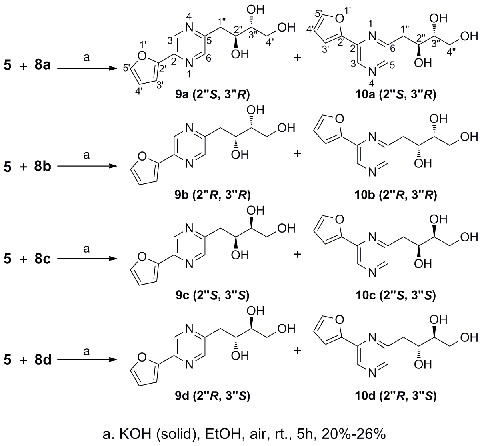

## Introduction

Pyrazines are important components of processed foods and are associated with the generation of flavor and color, such as those of fermented cacao, cheese and wines.[1] The Maillard reaction of amino acids with reducing sugars has become the primary process for generating these pyrazine derivatives in food processing.[2] Some molecules containing the pyrazine pharmacophore have exhibited remarkable pharmacological activities with antitumor,[3] diuretic,[4] immunomodulatory[5] and analgesic effects.[6] Crotonine (2-(furan-2-yl)-5-(2,3,4-trihydroxy-butyl)-1,4-diazine) was isolated from *Croton tiglium* L. (Euphoriaceae) without determination of the absolute configuration. However, the analgesic properties were obvious compared to those of morphine.[7] To determine the absolute structure of natural crotonine, a concise saccharide-based five-step route was developed for the synthesis of all possible chiral isomers. The synthesis of pyrazine derivatives has attracted much interest due to their numerous scientific applications in both chemistry and biology. The condensation reaction of diamines with diols,[8] hydroxyketones[9] and diketones[10] under catalysis by a transitional metal or base are the main reaction types reported in the literature. In our case, inexpensive monosaccharides were adopted as the starting material and chirality source for the preparation of $\alpha$-dicarbonyl intermediates.[11] Pyrazine condensation of the diamine and 3-deoxy-aldos-2-uloses intermediates, which is the key step in the reaction sequence, was catalyzed by potassium hydroxide, and other reaction conditions were successively investigated. The retrosynthetic analysis is outlined in Scheme 1.

*To whom correspondence should be addressed. E-mail: yangyangc@mail.kib.ac.cn

## Results and Discussion

The preparation of the 1,2-ethylenediamine intermediate **5** was initiated with furfuraldehyde **1** (Scheme 2).[12] Henry addition with nitromethane generated 2-(2-nitroethenyl)furan **2** in 72% yield, which was followed by treatment with methoxylamine hydrochloride to yield N-methoxyethylamine **3** in 88% yield. According to the published procedure, reduction of **3** by zinc powder and acetic acid was attempted to generate 1,2-ethylenediamine **5** directly, however, this reaction only yielded a series of partially reduced byproducts and was difficult for purification.[12] While catalytic hydrogenation over Pd/C only afforded partially reduced monoamine at the nitro group, when the Pd/C-catalyzed reaction was conducted under higher pressure or temperature, or a more active reducing reagent such as palladium hydroxide was used, the reduction of **3** produced an over-reduced product at the furan moiety. An exchange strategy succeeded in cleaving the methoxyl group from the nitrogen to yield nitro-ethenamine **4** in 95% yield,[13] followed by reduction with $LiAlH_4$ in THF to afford 1,2-ethylenediamine intermediate **5** in 52% yield.

Construction of the 3-deoxy-aldos-2-ulose intermediates **8a**–**8d** started with monosaccharides (Scheme 3), such as D-glucose **6a**, D-galactose **6b**, L-sorbose **6c** and L-glucose **6d**, which possess different potential chiral centers at the 2" and 3" positions in the final product. Refer to the method described in the literature,[11] the monosaccharides reacted with benzoyl hydrazine to yield 3-deoxy-bis(benzoylhydrazones) **7a**–**7d** in 63%–93% yield. The mechanism was explained as a repeated

**Scheme 1.** Retrosynthetic analysis of crotonine intramolecular rearrangement and dehydration process of the 1 and 2 position of the monosaccharide.[11] After easily obtaining the products **7a**–**7d** by filtration, a transhydrazonation reaction with benzaldehyde afforded 3-deoxy-aldos-2-uloses **8a**–**8d** in 25%–37% yield.

**Scheme 2.** Synthesis of diamine intermediate **5**

The condensation of the two key intermediates (i.e., **5** and **8a**–**8d**) was exhibited in Scheme 4, the reaction of **5** and **8** without base conducted under an argon atmosphere was unsuccessful,[14] because the alkalinity of **5** was insufficient to spontaneously catalyze the cyclization. When the strong base as potassium tert-butoxide or potassium hydroxide was used under an argon and anhydrous conditions,[15] a trace product was obtained, but the yield failed to increase even with a high temperature and long reaction time. Next, the reaction was attempted in an air atmosphere using potassium hydroxide at 0 ºC, but no reaction occurred.[16] Fortunately, the yield of **9** was improved after increasing the temperature to room temperature, but the regional isomers **10** were also produced, the quantity of product **9** and **10** were in a 1:1 ratio (the ratio was determined by $^1$H NMR analysis). After we screened the reaction conditions, including varying the quantity of the reagents and the addition method, it was determined that the addition frequency of potassium hydroxide markedly influenced the regioselectivity of the pyrazine condensation. In the synthesis of **9** and **10**, room temperature and multiple additions of potassium hydroxide (i.e., 10 portions of solid for 1 hour) favored the generation of a higher ratio of para-substitution **9** to meta-substitution **10** products (i.e., up to 5:1), and finally all 8 regional and chiral isomers were synthesized and purified successively.

A comparison of the spectroscopic data of the natural[7] and synthetic products was conducted (Tables 1 and 2). The $^1$H NMR of **10a**–**10d** differed from those of the natural product which exhibited a singlet peak for H-3 and H-5 of the pyrazine ring, indicating that **10a**–**10d** were meta-substitution products; while **9a**–**9d** and natural crotonine showed a doublet peak ($J$ = 1.1 to 1.4 Hz) which attributed them to the para-substitution products.[17] The remaining $^1$H and $^{13}$C NMR data of **10a**–**10d** were similar to that of **9a**–**9d,** inferring that each pair of **9** and **10** (i.e., **9a** and **10a**) were regional isomers and possessed the same chirality at 2" and 3" position which were controlled by the chirality of intermediates **8a**–**8d**. To the para-substitution products **9a**–**9d**, the characteristic proton signal of 1" of **9b** and **9c** crotonine showed only one set of doublet peak, that differed obviously from that of **9a**, **9d** and the natural crotonine which exhibited two set of dd peaks, in addition, the $^1$H and $^{13}$C NMR data for **9a** and **9d** matched those of the reported natural product exactly, indicating that the absolute configuration of natural crotonine only could be equal to **9a** or **9d**. A further comparison of the optical rotation of **9a** and **9d** indicated that that of **9a** (2"*S*, 3"*R*)-crotonine ($[\alpha]_D^{24}$ – 30.0 (*c* 0.17, MeOH)) was closer to that of the natural product ($[\alpha]_D^{20}$ – 6.8 (*c* 0.25, MeOH)), because the enantiomer **9d** (2"*R*, 3"*S*)-crotonine ($[\alpha]_D^{25}$ + 23.5 (*c* 0.18, MeOH)) produced an opposite optical rotation that was substantially different from that of the natural product. Therefore, we inferred that the most likely absolute configuration of crotonine was identical to **9a** as 2-(furan-2'-yl)-5-(2"*S*,3"*R*,4"-trihydroxy-butyl)-1,4-diazine.

In conclusion, we devised a concise five-step saccharide-based procedure to synthesize all eight possible regional and chiral isomers of crotonine for the first time. This method may be conveniently extended to other diamines and monosaccharides for the synthesis of a series of potentially bioactive pyrazines. Then, we determined that the most probable absolute configurations of the natural product by comparison of the NMR spectra and optical rotation data.

## Experimental Section

**General Experimental Procedures.** All reactions were performed with chemically pure solvents without further purifications unless otherwise noted. Dry solvent was obtained by a standard procedure. Dry tetrahydrofuran (THF) was distilled over a sodium-potassium alloy. Diethylformamide

**Scheme 3.** Synthesis of intermediates **8a**–**8d**

(DMF) was distilled over calcium hydride. Yields refer to chromatographically and spectroscopically homogeneous materials, unless otherwise noted. Reagents were used as received without further purification. Silica gel (200–300 mesh, Qingdao Marine Chemical Ltd., China), and light petroleum ether (bp 60–90 °C), ethyl acetate and methanol were used for product purification used for flash column chromatography. Purity of compounds were determined by Agilent 1200 Series HPLC using Thermo Hypersil Gold C18 column eluted with a mixture of methanol and water. NMR spectra were recorded in $CDCl_3$, $C_5D_5N$ and Methanol solutions on Bruker AV-400/800 and Bruker DRX-500 instrument with tetramethylsilane (TMS) as an internal reference. IR spectra were recorded with KBr pellets on a Bruker Tensor 27 FT-IR spectrometer. UV data were obtained on a Shimadzu UV-2401A spectrophotometer. Highresolution mass spectral analysis (HRMS) data were recorded via electron impact mass spectrometry using a time of flight analyzer. Optical rotations were determined on a Jasco P-1020 digital polarimeter.

**2-(2-Nitroethenyl)furan (2).** According to the literature procedure,[12] to a 2 L three neck flask was added **1** (100 g, 1.04 mol) and nitromethane (63.5 g, 1 equivalent) before 200 mL methanol was added at 0 °C, an aqueous sodium hydroxide (44 g, 1.1 equivalent) dissolved in 200 mL water was dropped into the mixture slowly to maintain the system stirring below 0 °C, then maintain the mixture stirring for 1 hour at 0 °C. Then 200 mL ice-water was poured into the mixture to generate a brown solution. To a cold solution prepared by 300 mL concentrated hydrochloride and 300 mL water, the former brown solution was added in slowly to furnish a yellow solid. Filtrated and washed by water several times, afforded the crude product, after repeated crystallization with methanol affording **2** as a yellow needle crystal 103 g (72%). $^1$H NMR (400 MHz, $CDCl_3$) $\delta$ ppm: 7.74 (d, $J$ = 13.2 Hz, 1H), 7.56 (dd, $J$ = 1.2, 0.5 Hz, 1H), 7.47 (d, $J$ = 13.2 Hz, 1H), 6.88 (d, $J$ = 3.5 Hz, 1H), 6.55 (dd, $J$ = 3.5, 1.8 Hz, 1H).

**1-(Furan-2-yl)-2-nitro-N-methoxyethylamine (3).** To a mixture of **2** (102 g, 0.73 mol), methoxylamine hydroxychloride (74 g, 1.2 equivalent) and sodium bicarbonate (74 g, 1.2 equivalent) was dissolved with 150 mL water and stirred, at the end of carbon dioxide generation, 650 mL THF was added and discharged the atmosphere with nitrogen for three times, stirred at room temperature overnight. When the reaction was complete, seperated 3 times by 200 mL ethyl acetate before combined the organic layer and concentrated the solvent, filtrated and affordded a yellow liquid 92 g (88%). $^1$H NMR (400 MHz, $CDCl_3$) $\delta$ ppm: 7.37 (m, 1H), 6.34 (m, 1H), 6.31 (d, $J$ = 2.5 Hz, 1H), 5.74 (br. s, 1H), 4.96–4.75 (m, 2H), 4.69 (dd, $J$ = 11.9, 4.2 Hz, 1H), 3.46 (s, 3H); $^{13}$C NMR (100 MHz, $CDCl_3$) $\delta$ ppm: 148.9, 142.6, 110.4, 108.4, 74.5, 62.4, 56.3.

**1-(Furan-2-yl)-2-nitro-ethenamine (4).** Added a solution of **3** in 50 mL dry DMF (22 g, 118.3 mmol) slowly to t-BuOK (29.2 g, 2.2 equivalent) dissolved in 200 mL anhydrous THF, maitaning the temperature at – 10ºC, then keep stirring for 5 h at room temprature, seperated by ethyl acetate and water for 5 times, combined the organic layer and concentrated the solvent after dried over with sodium sulfate, and the crude product was purified by recrystallization with methanol, dried under air to generate **4** as a brown solid 17.3 g (95%). $^1$H NMR (400 MHz, $CDCl_3$) $\delta$ ppm: 7.64 (d, $J$ = 1.1 Hz, 1H), 7.08 (s, 1H), 6.96 (d, $J$ = 3.6 Hz, 1H), 6.59 (dd, $J$ = 3.6, 1.7 Hz, 1H); $^{13}$C NMR (100 MHz, $CDCl_3$) $\delta$ ppm: 145.9, 145.1, 113.0, 112.8, 108.4; ESIMS: 155 $[M + H]^+$, 177 $[M + Na]^+$.

**1-(Furan-2-yl)-1,2-ethylenediamine (5)**. To a solution of $LiAlH_4$ in 200 mL dry THF was dropped in a solution of **4** in 50 mL THF at a liquid nitrogen-acetone bath maintaining the temprature below – 10 ºC, the reaction finished after 5 hours at 0 ºC, quenched with water very carefully, filtrated and affored a yellow solution, purified by RP-18 column and gave **5** as a brown liquid 11.2 g (90 mmol, 52%), conserved at – 20 ºC under nitrogen. $^1$H NMR (400 MHz, $CDCl_3$) $\delta$ ppm: 7.35 (m, 1H), 6.28 (m, 1H), 6.17 (m, 1H), 3.95 (m, 1H), 3.04 (dd, $J$ = 12.5, 4.2 Hz, 1H), 2.93 (dd, $J$ = 7.7, 3.8 Hz, 1H), 2.14 (br. s, 4H); $^{13}$C NMR (100 MHz, $CDCl_3$): $\delta$ ppm: 157.0, 141.7, 110.1, 105.1, 51.7, 46.7; HRMSEI $m/z$: calcd for $C_6H_{10}N_2O$ $[M]^+$: 126.0793, found: 126.0796.

**3-Deoxy-D-erythro-hexos-2-ulose bis(benzoylhydrazone) (7a).** Followed the methods of Khadem,[11e] to a solution of 600 mL ethanol and 120 mL water was added D-glucose **6a** (30 g, 0.17 mmol), benzoylhydrazine (46 g, 0.34 mmol) and pa. KOH (solid), EtOH, air, rt., 5h, 20%-26%

**Scheme 4.** Synthesis of **9** and **10**

toluidine (12 g, 0.11 mmol) followed by 50 mL acetic acid, refluxed for 7 hours. Filtrated afforded a white solid, washed by water and ethanol, then a crystallization with ethanol generated **7a** as a white needle 44.6 g (0.12 mmol, 71%). $[\alpha]_D^{24}$ + 4.8 (*c* 0.56, Pyridine); $^1$H NMR (400 MHz, $C_5D_5N$) $\delta$ ppm: 14.61 (s, 1H), 14.33 (s, 1H), 10.41 (s, 1H), 9.71 (m, 4H), 9.00–8.82 (m, 6H), 6.24 (br. s, 1H), 5.98–5.66 (m, 3H), 5.14 (br. s, 2H); $^{13}$C NMR (100 MHz, $C_5D_5N$) $\delta$ ppm: 167.3, 166.5, 158.2, 151.1, 136.1, 135.8, 134.3, 131.0, 130.8, 130.5, 130.2, 77.0, 75.1, 66.3, 32.6; HRMSEI *m/z*: calcd for $C_{20}H_{22}N_4O_5$ $[M]^+$: 398.1590, found 398.1568.

**3-Deoxy-D-threo-hexos-2-ulose bis(benzoylhydrazone) (7b).** The procedure was conducted as the synthesis of **7a**, D-galactose **6b** 10 g, benzoylhydrazine 15.4 and p-toluidine 4 g, generated **7b** as a white needle 15.57 g (80%). $[\alpha]_D^{24}$ + 47.0 (*c* 0.42, Pyridine); $^1$H NMR (400 MHz, $C_5D_5N$) $\delta$ ppm: 14.48 (s, 1H), 14.35 (s, 1H), 10.30 (s, 1H), 9.81 (br. d, *J* = 7.1 Hz, 2H), 9.69 (br. d, *J* = 7.7 Hz, 2H), 8.89 (m, 6H), 6.33 (br. s, 1H), 5.96–5.66 (m, 3H), 5.31–5.01 (m, 2H); $^{13}$C NMR (100 MHz, $C_5D_5N$) $\delta$ ppm: 167.1, 166.1, 157.6, 150.7, 136.3, 134.1, 130.9, 130.7, 130.4, 130.2, 77.2, 73.6, 66.4, 33.4; HRMSEI *m/z*: calcd for $C_{20}H_{22}N_4O_5$ $[M]^+$: 398.1590, found 398.1601.

**3-Deoxy-L-threo-hexos-2-ulose bis(benzoylhydrazone) (7c).** The procedure was conducted as the synthesis of **7a**, L-sorbose **6c** 10 g, benzoylhydrazine 15.4 g and p-toluidine 4 g, generated **7c** as a white needle 18.5 g (93%). $[\alpha]_D^{24}$ – 51.2 (*c* 0.54, Pyridine); $^1$H NMR (400 MHz, $C_5D_5N$) $\delta$ ppm: 14.48 (s, 1H), 14.35 (s, 1H), 10.31 (s, 1H), 9.81 (br. d, *J* = 7.1 Hz, 2H), 9.69 (br. d, *J* = 7.7 Hz, 2H), 8.89 (m, 6H), 6.33 (br. s, 1H), 5.87 (m, 2H), 5.72 (m, 1H), 5.36–5.09 (m, 2H); $^{13}$C NMR (100 MHz, $C_5D_5N$) $\delta$ ppm: 167.1, 166.3, 157.6, 150.7, 136.3, 134.1, 130.9, 130.8, 130.4, 130.2, 77.2, 73.6, 66.4, 33.4; HRMSEI *m/z*: calcd for $C_{20}H_{22}N_4O_5$ $[M]^+$: 398.1590, found 398.1592.

**3-Deoxy-L-erythro-hexos-2-ulose bis(benzoylhydrazone) (7d).** The procedure was conducted as the synthesis of **7a**, L-glucose **6d** 1 g, benzoylhydrazine 1.54 g and p-toluidine 0.4 g, generated **7d** as a white needle 1.25 g (63%). $[\alpha]_D^{24}$ – 3.4 (*c* 0.45, Pyridine); $^1$H NMR (400 MHz, $C_5D_5N$) $\delta$ ppm: 14.60 (s, 1H), 14.27 (s, 1H), 10.27 (s, 1H), 9.70 (br. d, *J* = 7.1 Hz, 2H), 9.62 (br. d, *J* = 7.4 Hz, 2H), 8.92–8.84 (m, 2H), 8.80 (m, 4H), 6.20 (br. s, 1H), 5.93–5.61 (m, 3H), 5.07 (br. s, 2H); $^{13}$C NMR (100 MHz, $C_5D_5N$) $\delta$ ppm: 167.2, 166.2, 157.8, 151.0, 136.1, 135.7, 134.3, 130.9, 130.7, 130.3, 130.0, 76.8, 75.0, 66.2, 32.4; HRMSEI *m/z*: calcd for $C_{20}H_{22}N_4O_5$ $[M]^+$: 398.1590, found 398.1588.

**3-Deoxy-D-erythro-hexos-2-ulose (8a).** Followed the methods of Khadem,[11d] to 300 mL methanol was added **7a** (20 g, 54 mmol) and 500 mL water, after acetic acid (12 mL) and benzaldehyde (32 mL) were added in, the mixture was to refluxed for 2 hours, when the hydrazone was completely dissovled, the reaction was contiued to refluxed for another 10 hours with vigrously. Filtrated and the solution was concentrated in vaccour, the residue was seperated with water and dichloromethane by 5 times, combined the aqueous part and evaperated the solvent, furnished **8a** as a yellow colloidal solid 3.2 g (32 mmol, 37%); $^1$H NMR (400 MHz, $CD_3OD$) $\delta$ 4.42 (br. s, 1H), 4.39 (br. s, 1H), 4.37 (br. s, 1H), 4.30 (br. s, 1H), 4.28 (br. s, 1H), 4.24 (d, *J* = 2.7 Hz, 1H), 4.18 (m, 1H), 4.05 (m, 1H), 4.00 (m, 1H), 3.97 (m, 1H), 3.90 (d, *J* = 2.8 Hz,

**Table 1. $^{1}$H NMR data of natural crotonine and synthetic products in $CD_3OD$**

| Pos. | crotonine[1] | 9a[1] | 9b[2] | 9c[2] | 9d[2] | 10a[2] | 10b[2] | 10c[2] | 10d[2] |
|---|---|---|---|---|---|---|---|---|---|
| 2 | | | | | | | | | |
| 3 | 8.88 (d, 1.4) | 8.82 (d, 1.4) | 8.90 (d, 1.1) | 8.92 (d, 1.1) | 8.90 (d, 1.4) | 8.79 (s) | 8.78 (s, 1H) | 8.78 (s, 1H) | 8.79 (s, 1H) |
| 5 | | | | | | 8.39 (s) | 8.39 (s, 1H) | 8.39 (s, 1H) | 8.38 (s, 1H) |
| 6 | 8.49 (d, 1.4) | 8.45(d, 1.4) | 8.51 (d, 1.1) | 8.53 (d, 1.1) | 8.50 (d, 1.4) | | | | |
| 3' | 7.15 (dd, 0.6, 3.4) | 7.11 (d, 3.4) | 7.18 (d, 2.9) | 7.20 (d, 3.0) | 7.17 (d, 3.4) | 7.25 (d, 3.4) | 7.23 (d, 3.3) | 7.23 (d, 3.3) | 7.24 (d, 3.4) |
| 4' | 6.61 (dd, 1.7, 3.4) | 6.57 (dd, 3.4, 1.7) | 6.63 (dd, 3.3, 1.7) | 6.65(dd, 3.3, 1.7) | 6.63 (dd, 3.4, 1.7) | 6.65 (dd, 3.3, 1.7) | 6.63 (dd, 3.3, 1.7) | 6.64 (dd, 3.3, 1.7) | 6.64 (dd, 3.3, 1.7) |
| 5' | 7.69 (dd, 0.6, 1.7) | 7.66 (dd, 1.7, 0.6) | 7.72 (br. d, 0.9) | 7.73 (br. d, 0.9) | 7.71 (d, 1.7) | 7.74 (br. d, 0.9) | 7.72 (br. s) | 7.72 (br. s) | 7.74 (br. d, 0.9) |
| 1" | 2.91 (dd, 14.2, 9.5)<br>3.21 (dd, 14.2, 3.1) | 2.89 (dd, 14.2, 9.4)<br>3.18 (dd, 14.2, 3.1) | 3.06 (d, 6.6) | 3.08 (d, 6.6) | 2.92 (dd, 14.2, 9.5)<br>3.23 (dd, 14.2, 3.1) | 2.95 (dd, 14.2, 9.4)<br>3.26 (dd, 14.2, 3.0) | 3.07 (d, 6.7) | 3.07 (d, 6.7) | 2.93 (dd, 14.2, 9.4)<br>3.25 (dd, 14.2, 3.0) |
| 2" | 3.95 (m) | 3.95 (m, 2H) | 4.09 (m) | 4.10 (m) | 3.97 (m) | 4.04 (m) | 4.14 (td, 6.7, 3.0) | 4.13 (m) | 4.03 (m) |
| 3" | 3.56 (m) | 3.53 (m) | 3.58 (m) | 3.59 (m) | 3.56 (m) | 3.60 (m) | 3.60 (td, 5.9, 3.2) | 3.60 (m) | 3.61 (m) |
| 4" | 3.62 (dd, 11.2, 6.4)<br>3.77 (dd, 11.2, 3.8) | 3.62 (dd, 11.2, 6.2)<br>3.76 (dd, 11.3, 3.8) | 3.64 (dd, 11.1, 6.5)<br>3.70 (dd, 11.1, 5.4) | 3.66 (dd, 11.1, 6.5)<br>3.70 (dd, 11.3, 5.4) | 3.63 (dd, 11.3, 6.3)<br>3.78 (dd, 11.3, 3.8) | 3.67 (dd, 11.4, 6.3)<br>3.82 (dd, 11.3, 3.9) | 3.71 (dd, 11.1, 5.4)<br>3.65 (dd, 11.1, 6.4) | 3.71 (dd, 11.1, 5.4)<br>3.65 (dd, 11.1, 6.4) | 3.64 (dd, 11.3, 6.3)<br>3.80 (dd, 11.4, 3.9) |

[1]Recorded at 400 MHz; [2]Recorded at 800 MHz; $\delta$ (ppm) and $J$ in Hz.

1H), 3.86 (m, 1H), 3.80 (m, 1H), 3.69 (m, 4H), 3.65 (m, 2H), 3.61 (m, 2H), 3.57 (m, 1H), 3.53 (m, 1H), 3.42 (dd, $J$ = 3.9, 1.7 Hz, 2H), 3.30 (dd, $J$ = 5.5, 3.9 Hz, 1H), 2.45 (m, 1H), 2.16 (m, 3H), 1.99 (m, 1H), 1.85 (m, 2H), 1.76 (m, 2H); $^{13}$C NMR (100 MHz, $CD_3OD$) $\delta$ 172.4, 172.0, 170.9, 169.7, 106.8, 106.7, 106.5, 106.2, 100.7, 100.1, 100.0, 99.9, 98.4, 98.3, 93.7, 93.3, 88.4, 88.3, 87.3, 87.2, 74.3, 74.0, 73.7, 73.6, 72.5, 72.2, 72.1, 72.0, 69.2, 68.8, 67.8,66.6, 65.3, 65.2, 65.1, 65.0, 63.9, 63.8, 63.2, 62.9, 62.8, 62.8, 60.3, 60.3, 55.7, 55.7, 55.7, 55.6, 42.89, 42.4, 42.1, 41.9, 38.6, 34.2, 33.0, 32.6. $[\alpha]_D^{24}$ + 11.0 ($c$ 0.38, $H_2O$); HRMSEI $m/z$: calcd for $C_6H_{10}O_5$ $[M]^+$: 162.0528, found: 162.0534.

**3-Deoxy-D-threo-hexos-2-ulose (8b).** The procedure was conducted as the synthesis of **8a**. **7b** 15.57 g, acetic acid (18.7 mL) and benzaldehyde (25 mL), afforded a yellow colloidal solid 1.96 g (25%); $^{1}$H NMR (400 MHz, $CD_3OD$) $\delta$ 4.44 (m, 1H), 4.41 (br. s, 0.5H), 4.39 (br. s, 0.5H), 4.34 (m, 0.5H), 4.32 (br. s, 1H), 4.30 (br. s, 1H), 4.26 (m, 0.5H), 4.19 (m, 1.5H), 4.07 (m, 1.5H), 4.02 (d, $J$ = 5.0 Hz, 1.5H), 4.00 (d, $J$ = 1.2 Hz, 1.5H), 3.88 (dd, $J$ = 4.5, 2.8 Hz, 1H), 3.83 (d, $J$ = 2.3 Hz, 0.5H), 3.80 (m, 0.5H), 3.72 (m, 5H), 3.67 (m, 2.5H), 3.62 (m, 1.5H), 3.59 (d, $J$ = 5.0 Hz, 1.5H), 2.46 (dt, $J$ = 13.7, 7.5 Hz, 1H), 2.17 (m, 3H), 2.02 (m, 0.6H), 1.82 (m, 4H); $^{13}$C NMR (150 MHz, $CD_3OD$) $\delta$ 172.4, 172.2, 171.7, 171.3, 105.5, 105.4, 105.2, 105.0, 99.5, 99.4, 98.8, 98.8, 98.7, 87.2, 86.0, 85.9, 71.3, 71.0, 70.8, 70.7, 68.0, 65.4, 63.9, 63.9, 62.6, 62.6, 61.9, 61.5, 41.6, 41.2, 40.9, 40.7, 37.4, 33.0, 31.8, 31.3; $[\alpha]_D^{24}$ – 19.8 ($c$ 0.41, $H_2O$); HRMSEI $m/z$: calcd for $C_6H_{10}O_5$ $[M]^+$: 162.0528, found: 162.0531.

**3-Deoxy-L-threo-hexos-2-ulose (8c).** The procedure was conducted as the synthesis of **8a**. **7c** 8.54 g, acetic acid 10.3 mL and benzaldehyde 13.7 mL, afforded **8c** as a yellow colloidal solid 1.07 g (31%); $^{1}$H NMR (400 MHz, $CD_3OD$) $\delta$ 4.44 (br. s, 1H), 4.41 (br. s, 0.5H), 4.39 (s, 0.5H), 4.30 (m, 1H), 4.32 (d, $J$ = 4.5 Hz, 1H), 4.29 (d, $J$ = 4.5 Hz, 1H), 4.27 (d, $J$ = 2.6 Hz, 1H), 4.19 (m, 0.5H), 4.06 (m, 1H), 4.02 (d, $J$ = 5.3 Hz, 1H), 4.00 (m, 1H), 3.88 (m, 1H), 3.83 (dd, $J$ = 5.0, 2.7 Hz, 1H), 3.80 (m, 1.5H), 3.70 (m, 3.5H), 3.63 (m, 2H), 3.56 (m, 2.5H), 2.46 (m, 1H), 2.15 (m, 2H), 2.02 (m, 0.5H), 1.81 (m, 4H); $^{13}$C NMR (100MHz, $CD_3OD$) $\delta$ 170.1, 170.0, 169.6, 169.3, 105.5, 105.4, 105.2, 105.0, 99.5, 99.5, 98.8, 98.8, 98.7, 87.2, 87.0, 86.0, 85.9, 71.3, 71.0, 70.8, 67.9, 65.4, 65.3, 64.0, 63.9, 62.6, 62.6, 61.9, 61.6, 41.6, 41.2, 40.9, 40.7, 35.2, 33.0, 31.8, 31.4; $[\alpha]_D^{24}$ + 12.2 ($c$ 0.55, $H_2O$); HRMSEI $m/z$: calcd for $C_6H_{10}O_5$ $[M]^+$: 162.0528, found: 162.0537.

**3-Deoxy-L-erythro-hexos-2-ulose (8d).** The procedure was conducted as the synthesis of **8a**. **7d** 1.15 g, acetic acid 1.38 mL and benzaldehyde 1.85 mL, afforded **8d** as a yellow colloidal solid 104 mg (23%); $^{1}$H NMR (400 MHz, $CD_3OD$) $\delta$ 4.44 (m, 1H), 4.41 (d, $J$ = 3.1 Hz, 0.5H), 4.39 (m, 0.5H), 4.32 (br. s, 1H), 4.30 (br. s, 1H), 4.20 (m, 1H), 4.08 (dt, $J$ = 9.4, 4.2 Hz, 1.5H), 4.02 (dd, $J$ = 6.5, 3.7 Hz, 2H), 4.00 (d, $J$ = 4.5 Hz, 2H), 3.89 (m, 1H), 3.72 (m, 5H), 3.68 (m, 3H), 3.62 (m, 3H), 2.45 (m, 1H), 2.18 (m, 2.5H), 1.84 (m, 5H); $^{13}$C NMR (100 MHz, $CD_3OD$) $\delta$ 171.2, 170.4, 170.2, 169.8, 105.6, 105.4, 105.3, 105.0, 99.5, 99.5, 98.9, 98.8, 98.7, 87.1, 87.0, 86.0, 85.9, 71.3, 70.9, 71.0 , 67.9, 65.4, 65.4, 63.9, 62.7, 62.6, 61.6 54.5, 54.5, 54.4, 41.6, 41.2, 40.9, 40.7, 33.1, 31.8, 31.4; $[\alpha]_D^{24}$ – 10.2 ($c$ 0.62, $H_2O$); HRMSEI $m/z$: calcd for $C_6H_{10}O_5$ $[M]^+$: 162.0528, found: 162.0529.

**Synthesis of 9 and 10.** To 1.5 equivalent **8** dissolved in ethaol was added 1.0 equivalent **5** in ethanol, allowed the mixture to stirred for 30 miniuts before pattasium hydroxide (3.0 equivalent) was added in 10 portions for 1 hour at room temperature, and kept reaction for a further 5 hours. Quanched by saturated ammonium chloride aqueous solution and evaporated the ethanol, after 20 mL water was added, extracted the low polarity impurities with dichloromethane for one time, the aquous phase was extrated with n-bultyl alchohol for 5 times, combined the organic phase and concentrated under vaccuum, seperated the product by silica gel column and obtained the mixed **9** and **10** isomers, after seperating by repeated Preparative Thin-Layer Chromatography with an

**Table 2.** $^{13}$C NMR data of crotonine and synthetic products in $CD_3OD$

| Position | crotonine | 9a[1] | 9b[2] | 9c[2] | 9d[2] | 10a[2] | 10b[2] | 10c[2] | 10d[2] |
|---|---|---|---|---|---|---|---|---|---|
| 2 | 143.9 | 143.8 | 144.0 | 143.9 | 144.0 | 145.6 | 145.7 | 145.9 | 145.6 |
| 3 | 140.1 | 140.1 | 140.2 | 140.1 | 140.1 | 138.2 | 138.2 | 138.4 | 138.1 |
| 5 | 154.8 | 154.7 | 154.6 | 154.5 | 154.8 | 144.1 | 144.0 | 144.2 | 144.1 |
| 6 | 145.7 | 145.7 | 145.9 | 145.8 | 145.8 | 156.7 | 156.5 | 156.7 | 156.7 |
| 2' | 152.3 | 152.2 | 152.4 | 152.3 | 152.4 | 152.5 | 152.5 | 152.7 | 152.5 |
| 3' | 111.2 | 111.3 | 111.4 | 111.2 | 111.3 | 111.9 | 111.9 | 112.1 | 111.9 |
| 4' | 113.3 | 113.3 | 113.4 | 113.2 | 113.3 | 113.4 | 113.4 | 113.6 | 113.4 |
| 5' | 145.9 | 145.9 | 145.8 | 145.7 | 146.0 | 146.0 | 146.0 | 146.2 | 146.0 |
| 1" | 39.6 | 39.6 | 40.0 | 39.9 | 39.7 | 39.8 | 40.2 | 40.4 | 39.8 |
| 2" | 72.9 | 72.9 | 72.2 | 72.1 | 73.0 | 72.9 | 72.1 | 72.3 | 73.0 |
| 3" | 76.1 | 76.1 | 75.1 | 75.1 | 76.2 | 76.2 | 75.2 | 75.4 | 76.2 |
| 4" | 64.6 | 64.5 | 64.4 | 64.3 | 64.6 | 64.6 | 64.4 | 64.6 | 64.6 |

[1]Recorded at 400 MHz; [2]Recorded at 800 MHz; $\delta$ (ppm) and $J$ in Hz.

eluent (ethyl acetate/methanol/aqueous ammonia = 98:2:5) and affordded the final sturcture **9** and **10** successively.

**Synthesis of 9a and 10a**. 500 mg **5**, 964 mg **8a** and 666 mg pattasium hydroxide followed the general procedure A and afforded 261 mg yellow solid (26%), took out 60 mg and seperated by PTLC, obtained **9a** (11.5 mg) and **10a** (14 mg) as yellow solid successively.

**2-(Furan-2'-yl)-5-(2"*S*,3"*R*,4"-trihydroxy-butyl)-1,4-diazine (9a).** $^1$H and $^{13}$C NMR see Tables 1 and 2; $[\alpha]_D^{24}$ – 30.0 ($c$ 0.17, MeOH); UV (MeOH) $\lambda_{max}$(log ε): 272 (4.17) nm; IR (KBr) $\nu_{max}$ cm$^{-1}$: 3424, 2925, 1630, 1498, 1033, 884, 745, 591; $^1$H and $^{13}$C NMR data, see Tables 1 and 2; HREIMS $m/z$: calcd for $C_{12}H_{14}N_2O_4$ $[M]^+$: 250.0954, found: 250.0951.

**2-(Furan-2'-yl)-6-(2"*S*,3"*R*,4"-trihydroxy-butyl)-1,4-diazine (10a).** $^1$H and $^{13}$C NMR see Tables 1 and 2; $[\alpha]_D^{25}$ – 60.3 ($c$ 0.09, MeOH); UV (MeOH) $\lambda_{max}$(log ε): 328 (4.15) nm; IR (KBr) $\nu_{max}$ cm$^{-1}$: 3406, 2923, 2884, 1630, 1608, 1531, 1491, 1154, 1068, 884, 749, 593; $^1$H and $^{13}$C NMR data, see Tables 1 and 2; HREIMS $m/z$: calcd for $C_{12}H_{14}N_2O_4$ $[M]^+$: 250.0954, found: 250.0961.

**Synthesis of 9b and 10b**. 50 mg **5**, 95 mg **8b** and 66 mg pattasium hydroxide followed the general procedure and afforded 23 mg yellow solid (25%), seperated by PTLC and obtained **9b** (3.3 mg) and **10b** (10 mg) as yellow solid successively.

**2-(Furan-2'-yl)-5-(2"*R*,3"*R*,4"-trihydroxy-butyl)-1,4-diazine (9b).** $^1$H and $^{13}$C NMR see Table 1 and Table 2; $[\alpha]_D^{24}$ + 58.7 ($c$ 0.07, MeOH); UV (MeOH) $\lambda_{max}$(log ε): 272 (4.14) nm; IR (KBr) $\nu_{max}$ cm$^{-1}$: 3421, 2925, 1631, 1501, 1384, 1113, 1077, 1037, 746, 593; $^1$H and $^{13}$C NMR data, see Tables 1 and 2; HREIMS $m/z$: calcd for $C_{12}H_{14}N_2O_4$ $[M]^+$: 250.0954, found: 250.0964.

**2-(Furan-2'-yl)-6-(2"*R*,3"*R*,4"-trihydroxy-butyl)-1,4-diazine (10b).** $^1$H and $^{13}$C NMR see Tables 1 and 2; $[\alpha]_D^{25}$ + 44.1 ($c$ 0.15, MeOH); UV (MeOH) $\lambda_{max}$(log ε): 328 (4.70) nm; IR (KBr) $\nu_{max}$ cm$^{-1}$: 3421, 2956, 2927, 1630, 1605, 1529, 1491, 1408, 1153, 1091, 1039, 1018, 752; $^1$H and $^{13}$C NMR data, see Tables 1 and 2; HREIMS $m/z$: calcd for $C_{12}H_{14}N_2O_4$ $[M]^+$: 250.0954, found: 250.0950.

**Synthesis of 9c and 10c**. 50 mg **5**, 95 mg **8c** and 66 mg pattasium hydroxide followed the general procedure and afforded a 19 mg yellow solid (20%), seperated by PTLC and obtained **9c** (1.5 mg) and **10c** (6.4 mg) as yellow solid successively.

**2-(Furan-2'-yl)-5-(2"*S*,3"*S*,4"-trihydroxy-butyl)-1,4-diazine (9c).** $^1$H and $^{13}$C NMR see Tables 1 and 2; $[\alpha]_D^{25}$ – 50.4 ($c$ 0.18, MeOH); UV (MeOH) $\lambda_{max}$(log ε): 273 (4.03) nm; IR (KBr) $\nu_{max}$ cm$^{-1}$: 3423, 2928, 1630, 1604, 1501, 1111, 1059, 1033, 751, 593; $^1$H and $^{13}$C NMR data, see Tables 1 and 2; HREIMS $m/z$: calcd for $C_{12}H_{14}N_2O_4$ $[M]^+$: 250.0954, found: 250.0958.

**2-(Furan-2'-yl)-6-(2"*S*,3"*S*,4"-trihydroxy-butyl)-1,4-diazine (10c).** $^1$H and $^{13}$C NMR see Tables 1 and 2; $[\alpha]_D^{24}$ – 36.9 ($c$ 0.12, MeOH); UV (MeOH) $\lambda_{max}$(log ε): 328 (3.96) nm IR (KBr) $\nu_{max}$ cm$^{-1}$: 3423, 2927, 1630, 1529, 1491, 1153, 1091, 1039, 1018, 752, 595; $^1$H and $^{13}$C NMR data, see Tables 1 and 2; HREIMS $m/z$: calcd for $C_{12}H_{14}N_2O_4$ $[M]^+$: 250.0954, found: 250.0958.

**Synthesis of 9d and 10d.** 30 mg **5**, 58 mg **8d** and 40 mg pattasium hydroxide followed the general procedure and afforded 13 mg yellow solid (22%), seperated by PTLC and obtained **9d** (1.5 mg) and **10d** (1.8 mg) as yellow solid successively.

**2-(Furan-2'-yl)-5-(2"*R*,3"*S*,4"-trihydroxy-butyl)-1,4-diazine (9d).** $^1$H and $^{13}$C NMR see Tables 1 and 2; $[\alpha]_D^{25}$ + 23.5 ($c$ 0.18, MeOH); UV (MeOH) $\lambda_{max}$(log ε): 272 (4.27) nm; IR (KBr) $\nu_{max}$ cm$^{-1}$: 3423, 2926, 1630, 1498, 1135, 1109, 1033, 746, 591; $^1$H and $^{13}$C NMR data, see Tables 1 and 2; HREIMS $m/z$: calcd for $C_{12}H_{14}N_2O_4$ $[M]^+$: 250.0954, found: 250.0948.

**2-(Furan-2'-yl)-6-(2"*R*,3"*S*,4"-trihydroxy-butyl)-1,4-diazine (10d).** $^1$H and $^{13}$C NMR see Tables 1 and 2; $[\alpha]_D^{25}$ + 65.3 ($c$ 0.24, MeOH); UV (MeOH) $\lambda_{max}$(log ε): 328 (3.95) nm; IR (KBr) $\nu_{max}$ cm$^{-1}$: 3417, 2922, 2883, 1631, 1531, 1491, 1409, 1154, 1068, 1038, 1015, 884, 749, 593; $^1$H and $^{13}$C NMR data, see Tables 1 and 2; HREIMS *m/z*: calcd for $C_{12}H_{14}N_2O_4$ $[M]^+$: 250.0954, found: 250.0961.

## Electronic Supplementary Material

Supplementary material is available in the online version of this article at http://dx.doi.org/10.1007/s13659-013-0080-1 and is accessible for authorized users.

## Acknowledgments

This work was supported financially and inspired scientifically by Prof. Yongxian Cheng of State Key Laboratory of Phytochemistry and Plant Resources in West China, Kunming Institute of Botany, Chinese Academy of Sciences, and Prof. Baomin Fan of Key Laboratory of Chemistry in Ethnic Medicinal Resources, State Ethnic Affairs Commission & Ministry of Education, Yunnan University of Nationalities.

**Open Access** This article is distributed under the terms of the Creative Commons Attribution License which permits any use, distribution, and reproduction in any medium, provided the original author(s) and source are credited.

## Electronic supplementary material

Supplementary material, approximately 2.73 MB.
