# Supplementary material for: Total synthesis and determination of the absolute configuration of a natural analgesic: crotonine
Source: Nat Prod Bioprospect. 2013 Dec 6;3(6):288–94. doi: 10.1007/s13659-013-0080-1 (PMC4131603; doi:10.1007/s13659-013-0080-1)

Electronic Supplementary Material

Total synthesis and determination of the absolute configuration of a natural analgesic: crotonine

Yang YANG[a,b,*]

[a]State Key Laboratory of Phytochemistry and Plant Resources in West China, Kunming Institute of Botany, Chinese Academy of Sciences, Kunming 650201, China

[b]Graduate University of Chinese Academy of Sciences, Beijing 100049, China

Received 9 October 2013; Accepted 21 November 2013

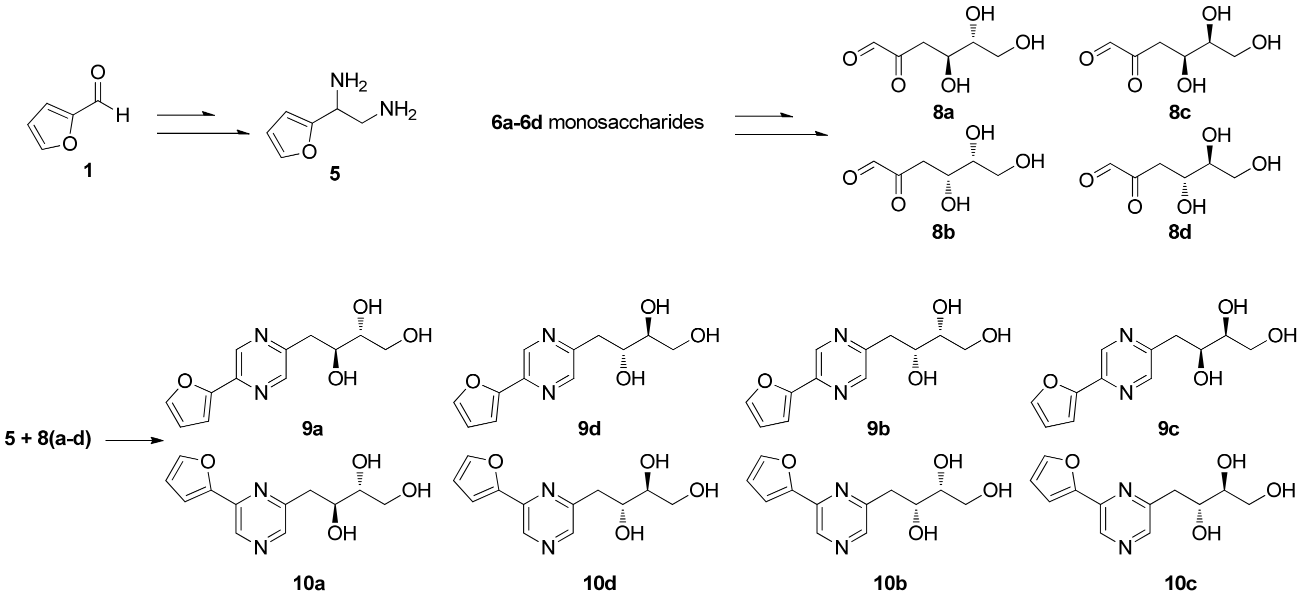

*To whom correspondence should be addressed. E-mail: yangyangc@mail.kib.ac.cn

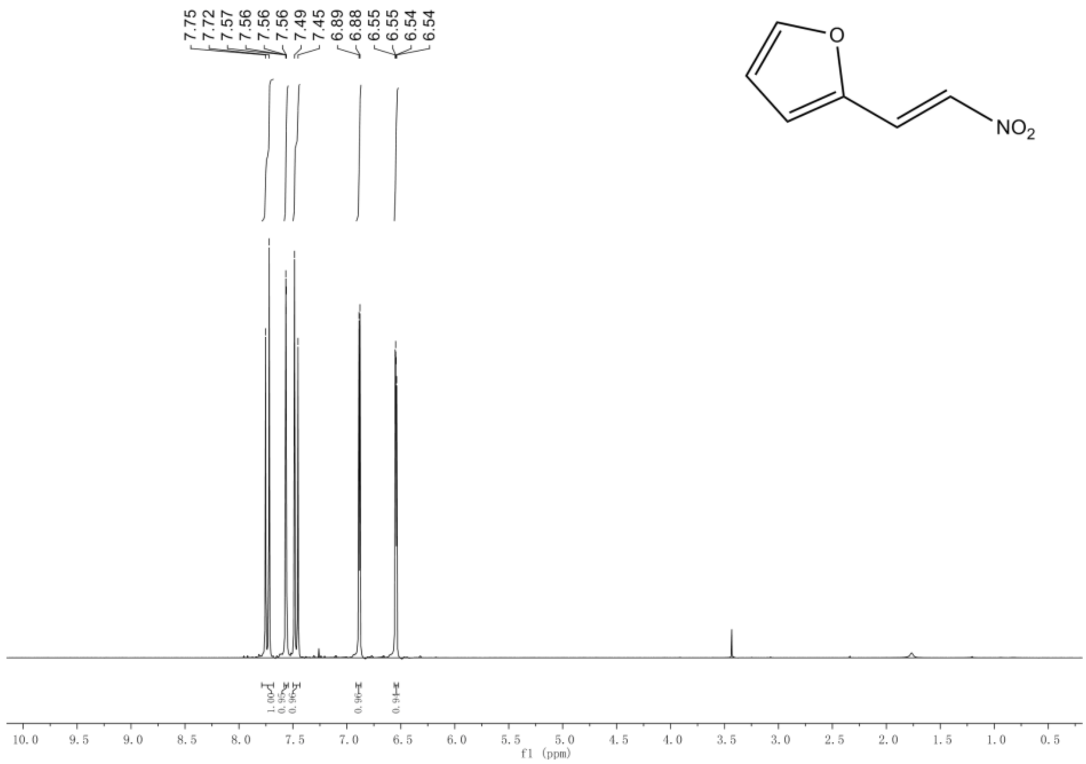
7.75
7.72
7.57
7.56
7.56
7.56
7.49
7.45
6.89
6.88
6.55
6.55
6.54
6.54
O
NO2
1.00
0.95
0.96
0.96
0.94
10.0 9.5 9.0 8.5 8.0 7.5 7.0 6.5 6.0 5.5 5.0 4.5 4.0 3.5 3.0 2.5 2.0 1.5 1.0 0.5
f1 (ppm)

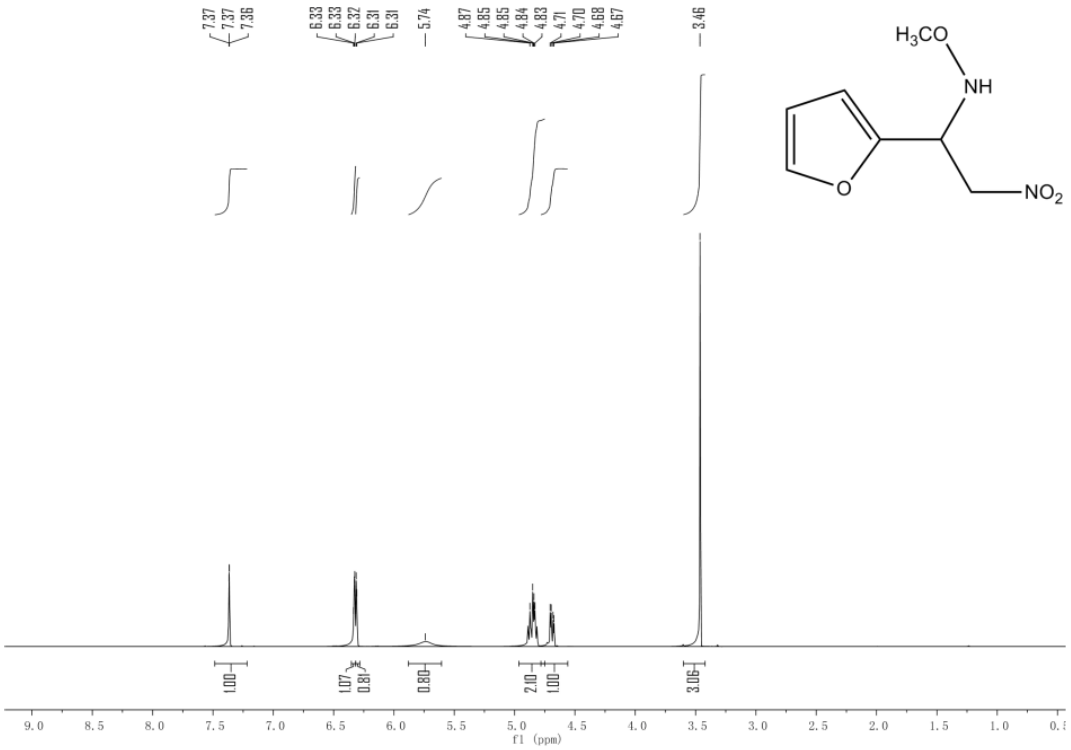

7.37
7.37
7.36
6.33
6.33
6.32
6.31
6.31
5.74
4.87
4.85
4.85
4.84
4.83
4.71
4.70
4.68
4.67
3.46
H3CO
NH
O
NO2
1.00
1.07
0.81
0.80
2.10
1.00
3.06
f1 (ppm)

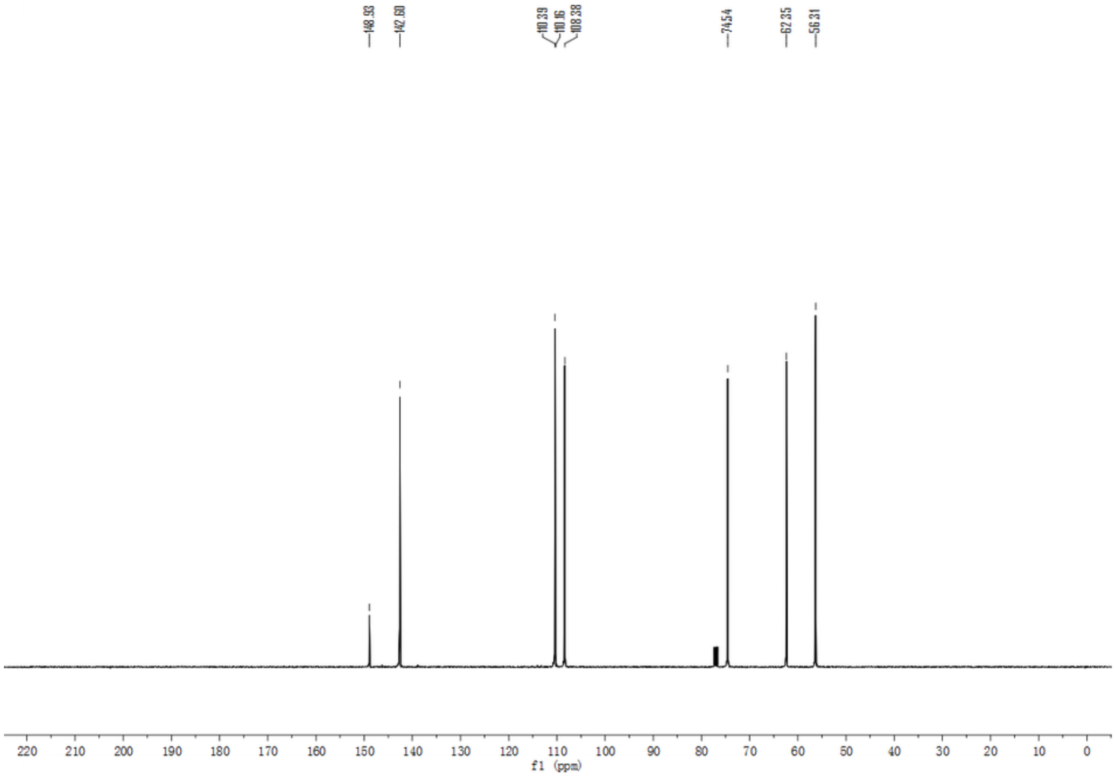

148.93
142.60
110.39
110.16
108.38
74.54
62.35
56.31
f1 (ppm)

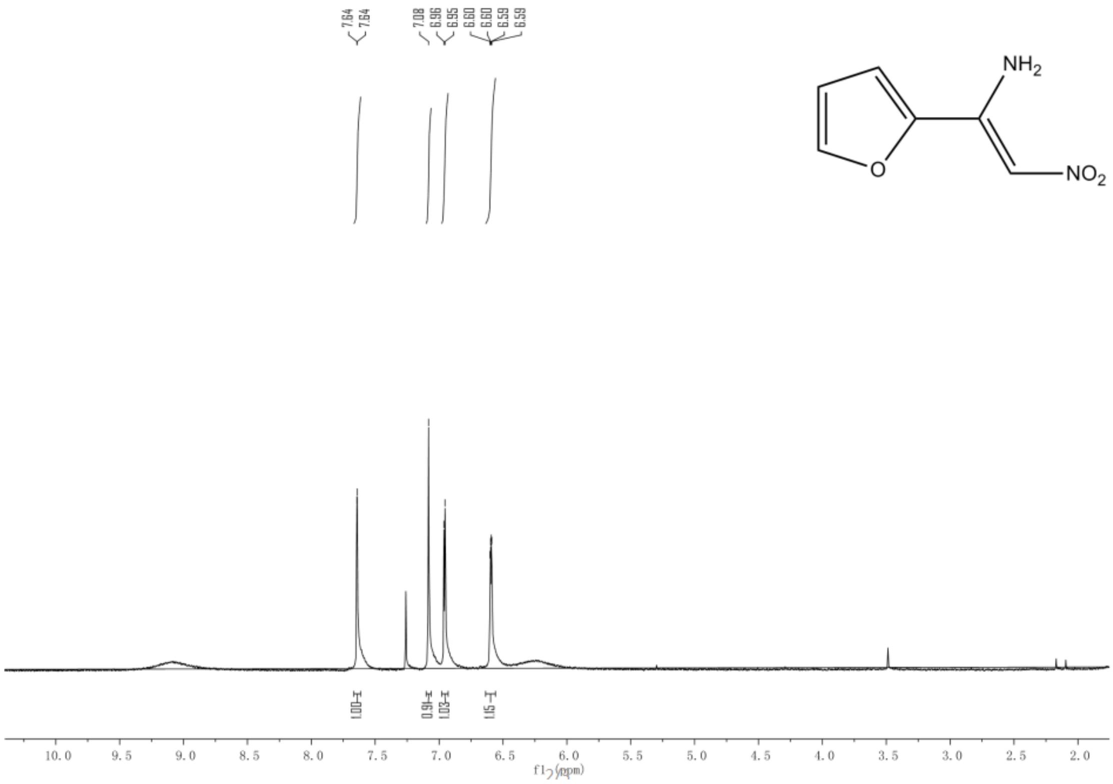
7.64
7.64
7.08
6.96
6.95
6.60
6.60
6.59
6.59
NH2
NO2
O
1.00
0.91
1.03
1.15
10.0 9.5 9.0 8.5 8.0 7.5 7.0 6.5 6.0 5.5 5.0 4.5 4.0 3.5 3.0 2.5 2.0
f1 (ppm)

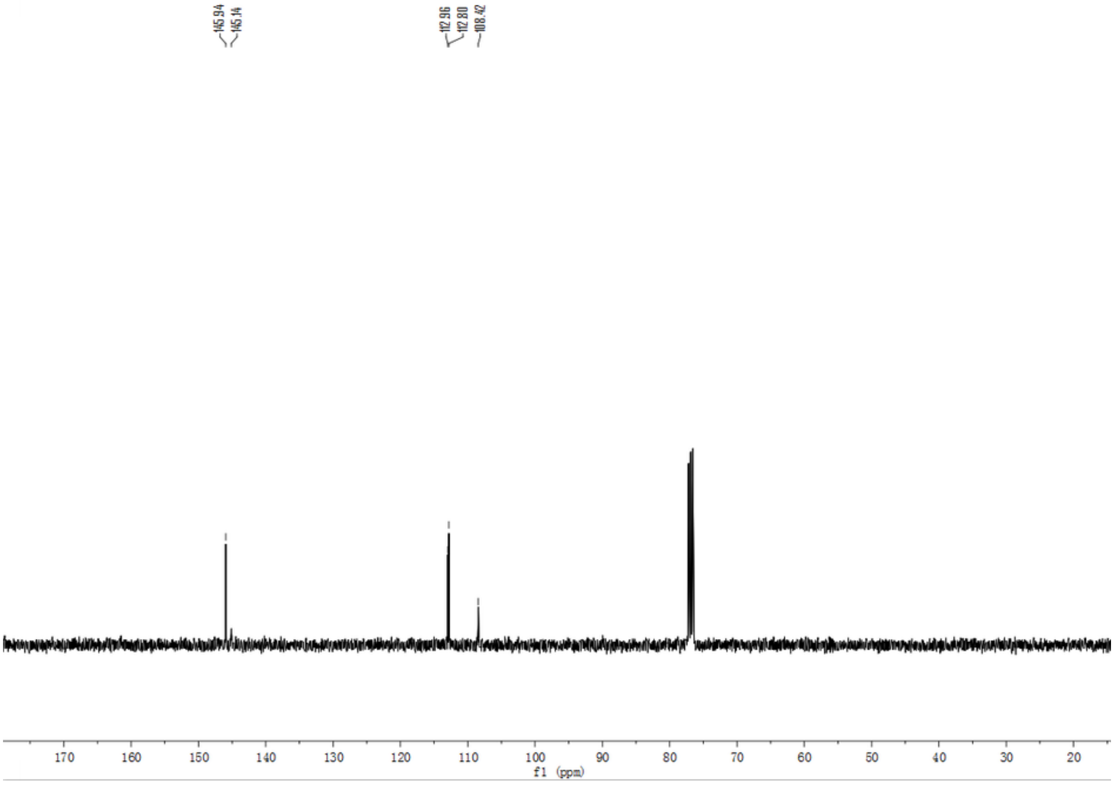
145.94
145.14
112.96
112.80
108.42
170 160 150 140 130 120 110 100 90 80 70 60 50 40 30 20
f1 (ppm)

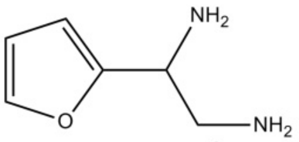
NH2
NH2
O

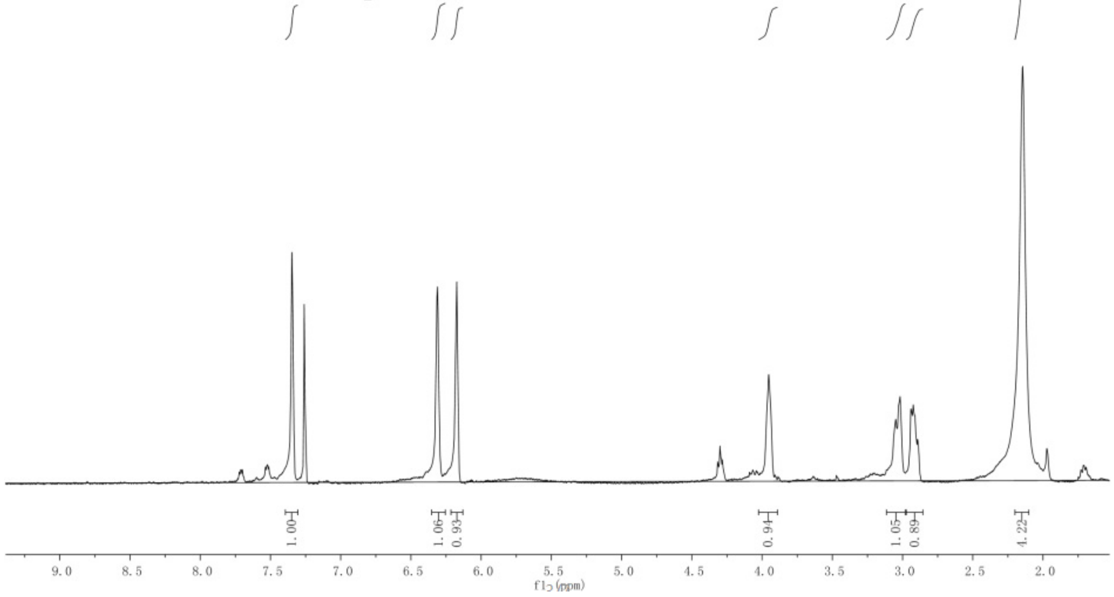
1.00
1.06
0.93
0.94
1.05
0.89
4.22
9.0 8.5 8.0 7.5 7.0 6.5 6.0 5.5 5.0 4.5 4.0 3.5 3.0 2.5 2.0
f1 (ppm)

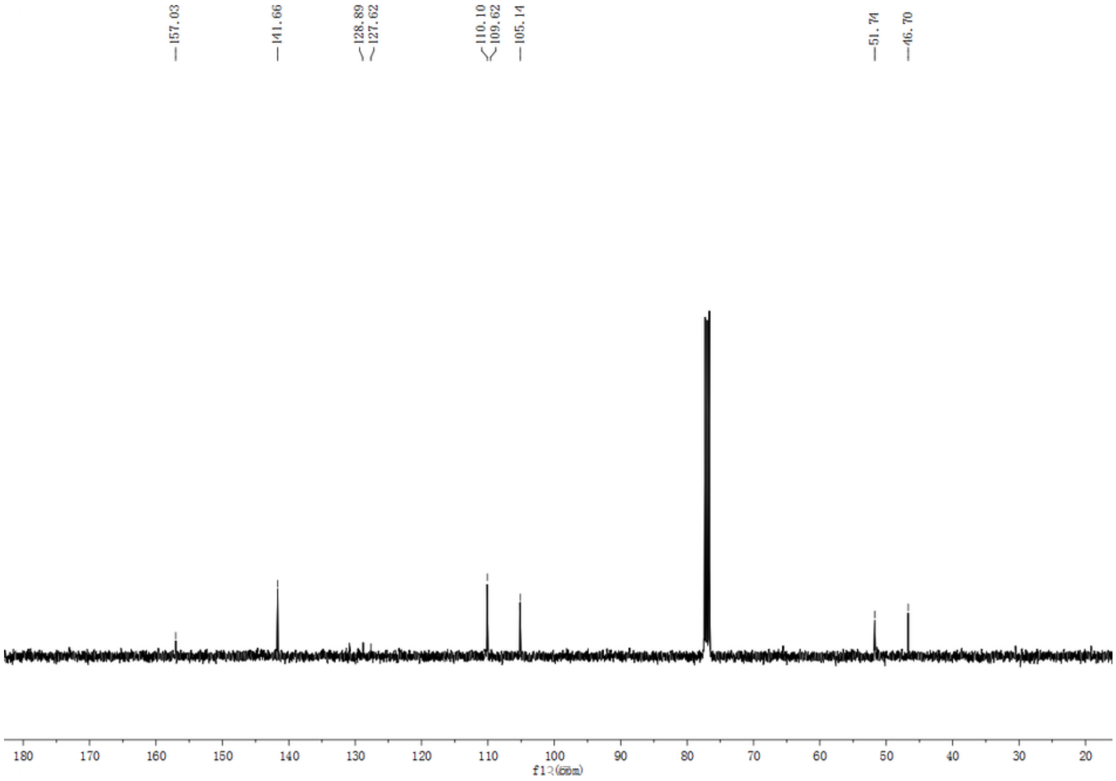
157.03
141.66
128.89
127.62
110.10
109.62
105.14
51.74
46.70
180 170 160 150 140 130 120 110 100 90 80 70 60 50 40 30 20

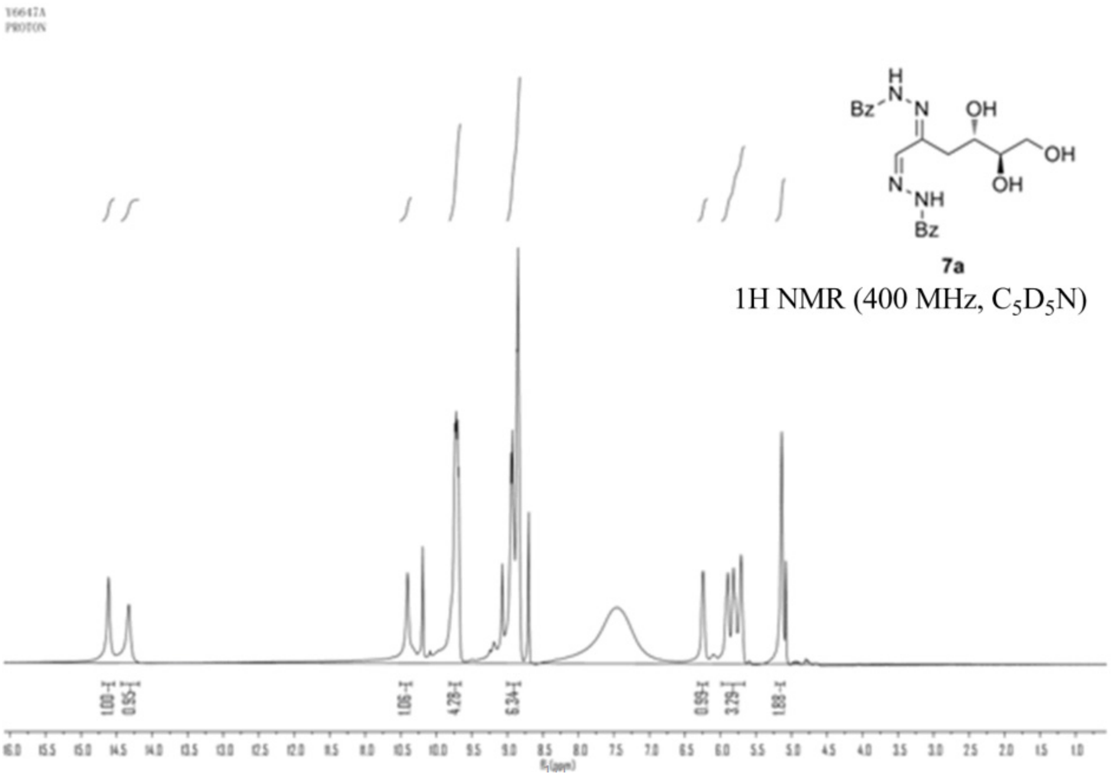

7a

1H NMR (400 MHz, C_5D_5N)

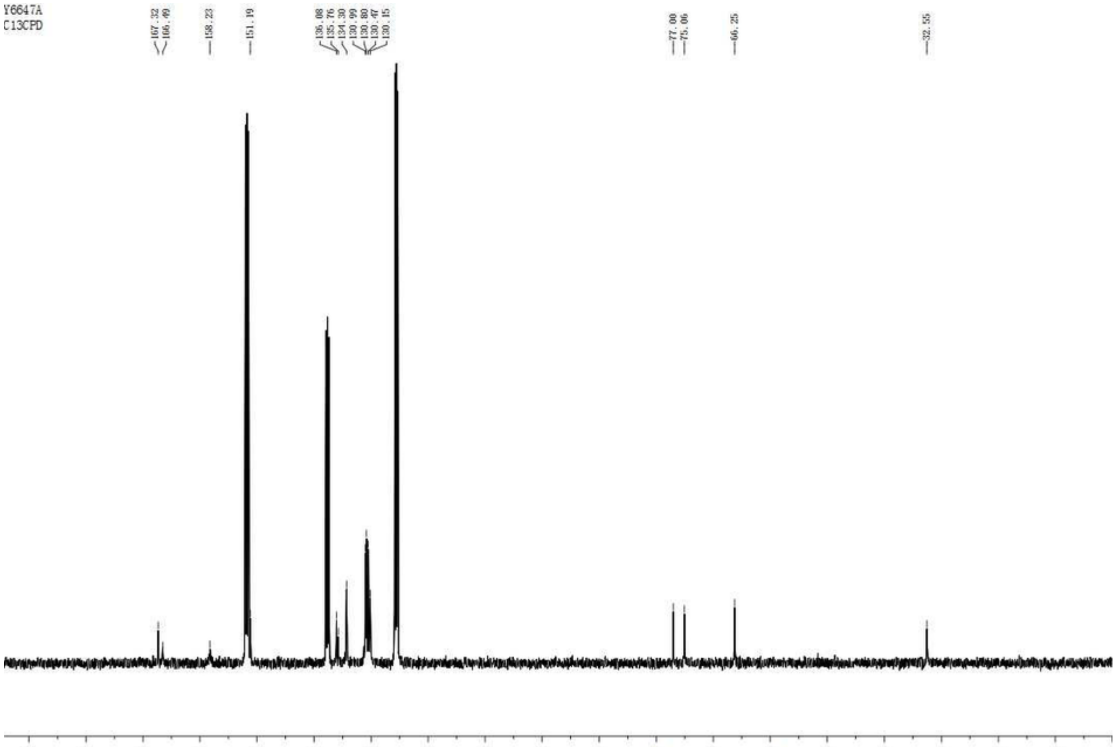

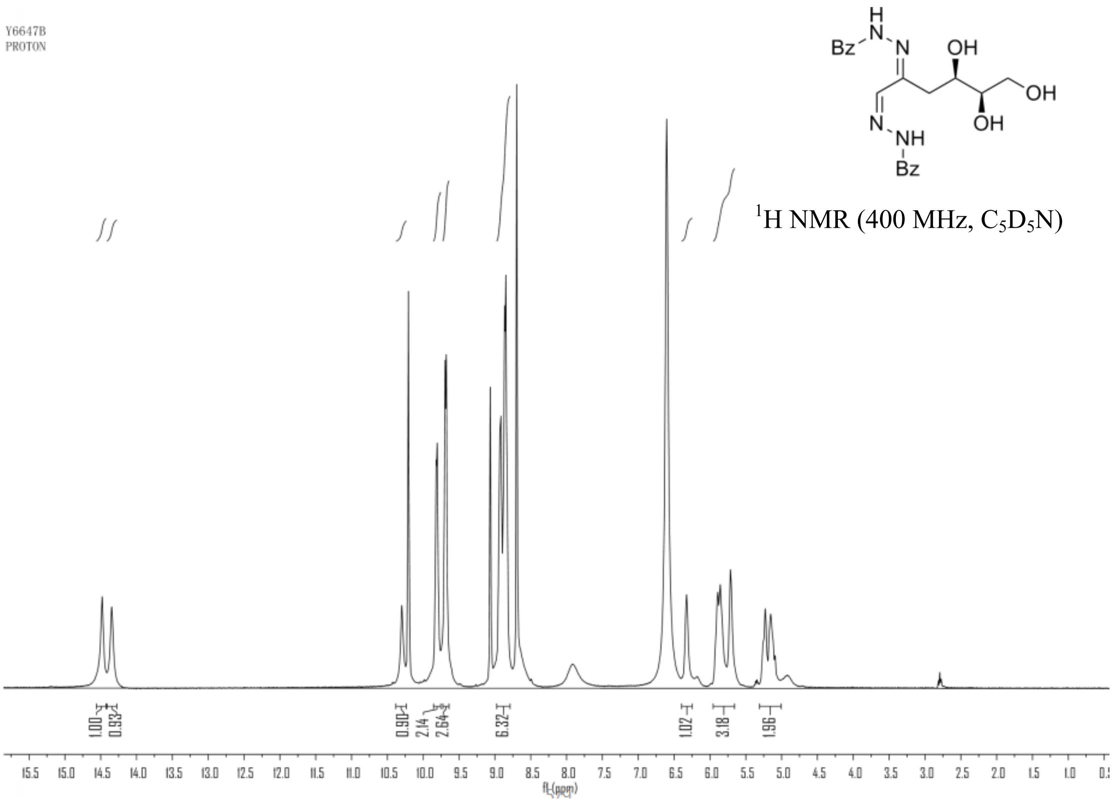

^{1}H NMR (400 MHz, C_5D_5N)

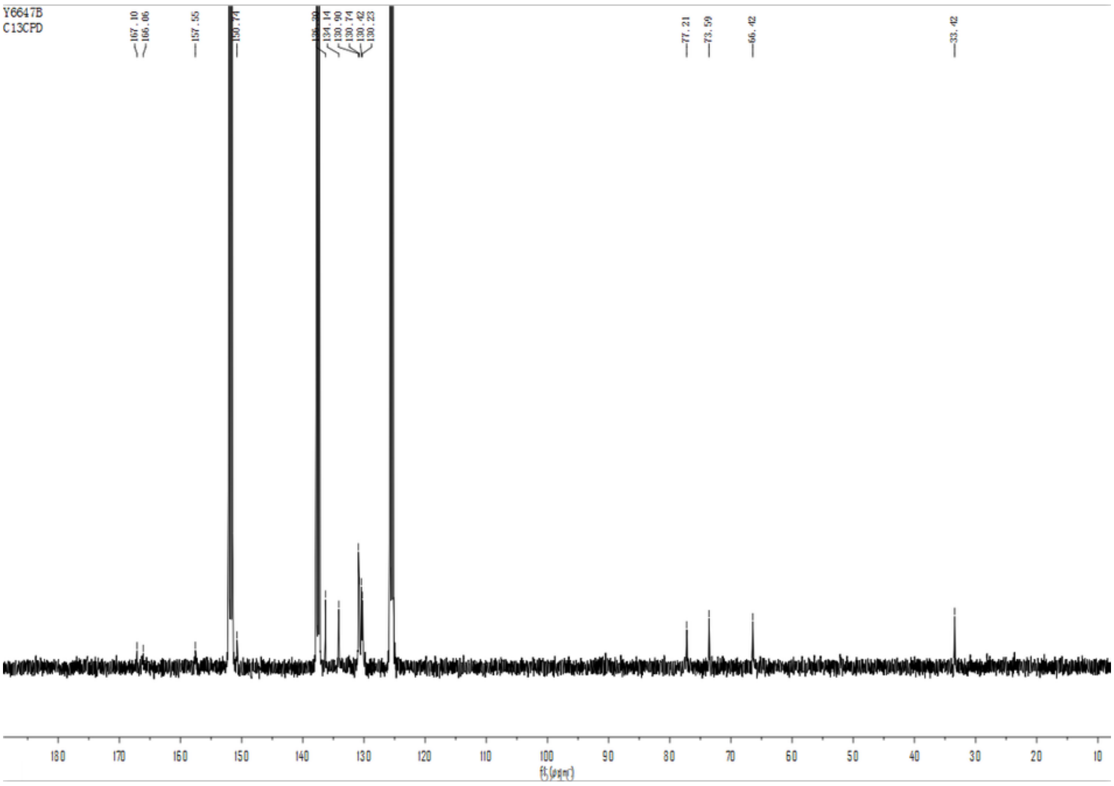

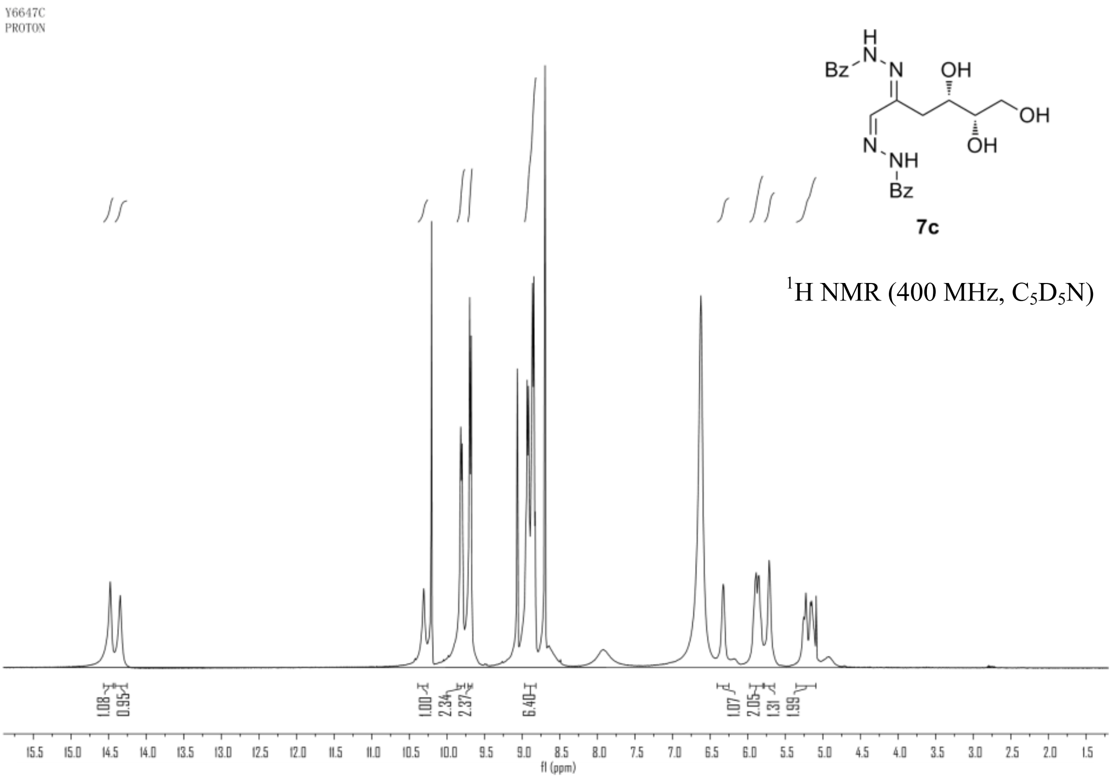

7c

^{1}H NMR (400 MHz, C_5D_5N)

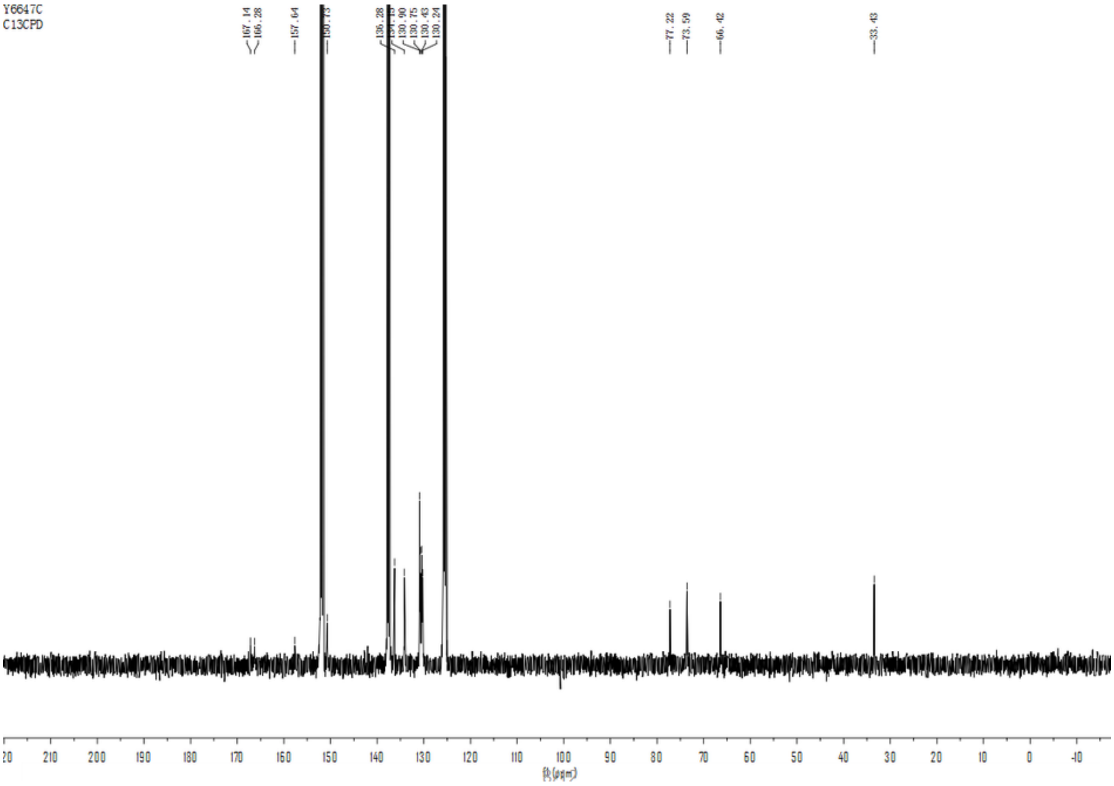

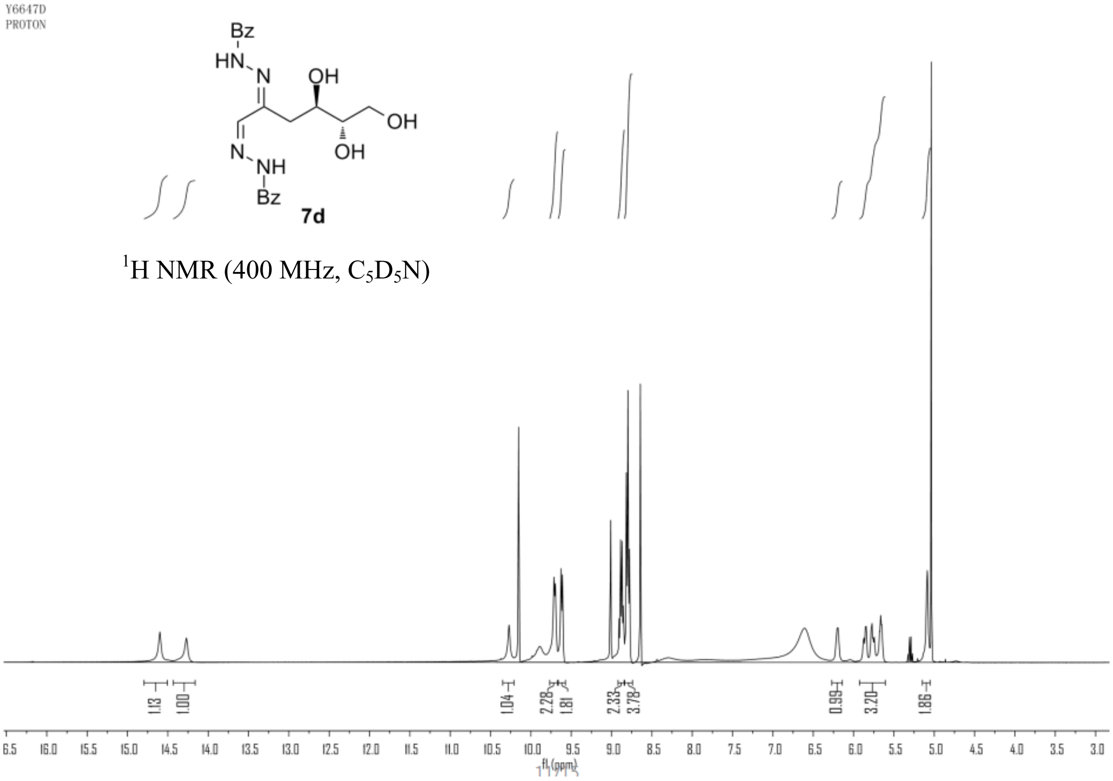

^{1}H NMR (400 MHz, C_5D_5N)

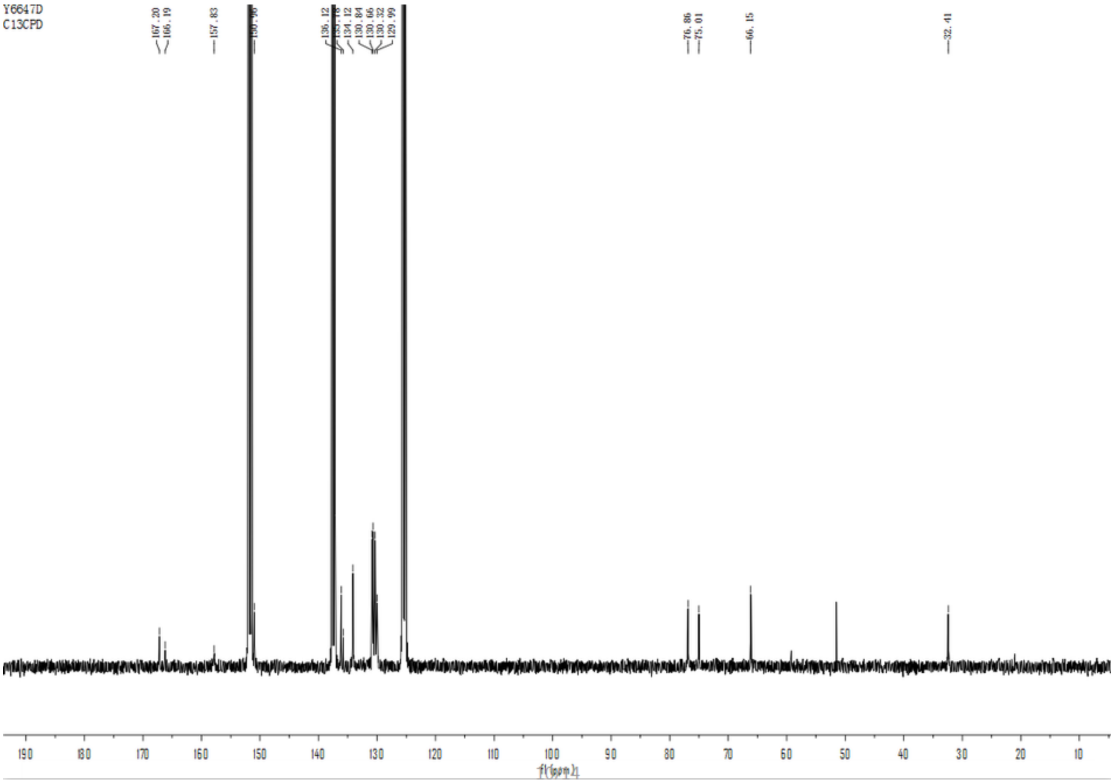

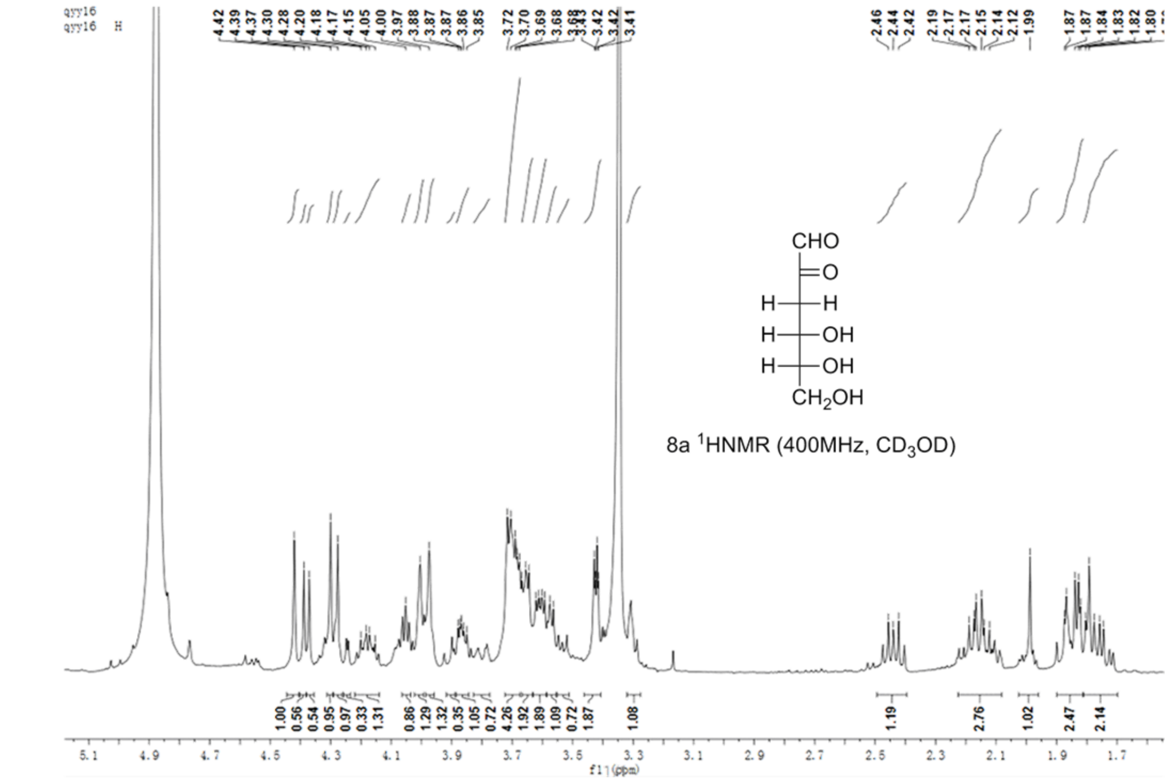
qyy16
qyy16 H
CHO
O
H
H
H
OH
H
OH
CH2OH
8a 1HNMR (400MHz, CD3OD)
f1 (ppm)

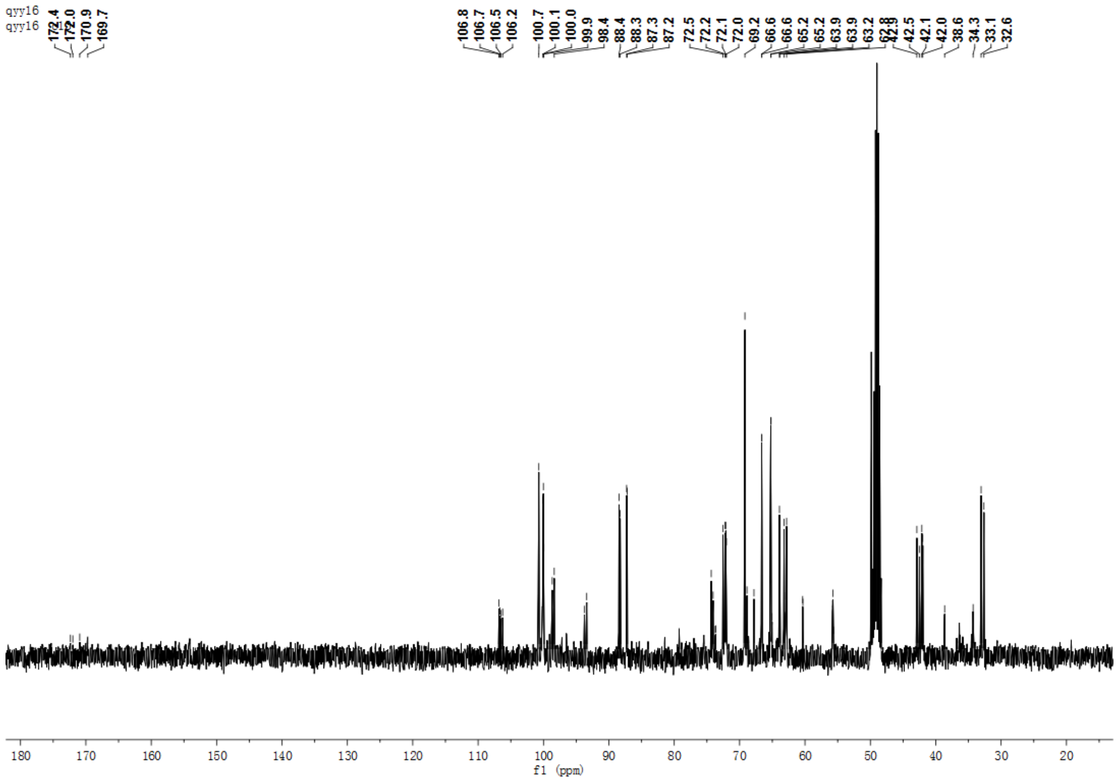
qyy16
qyy16
f1 (ppm)

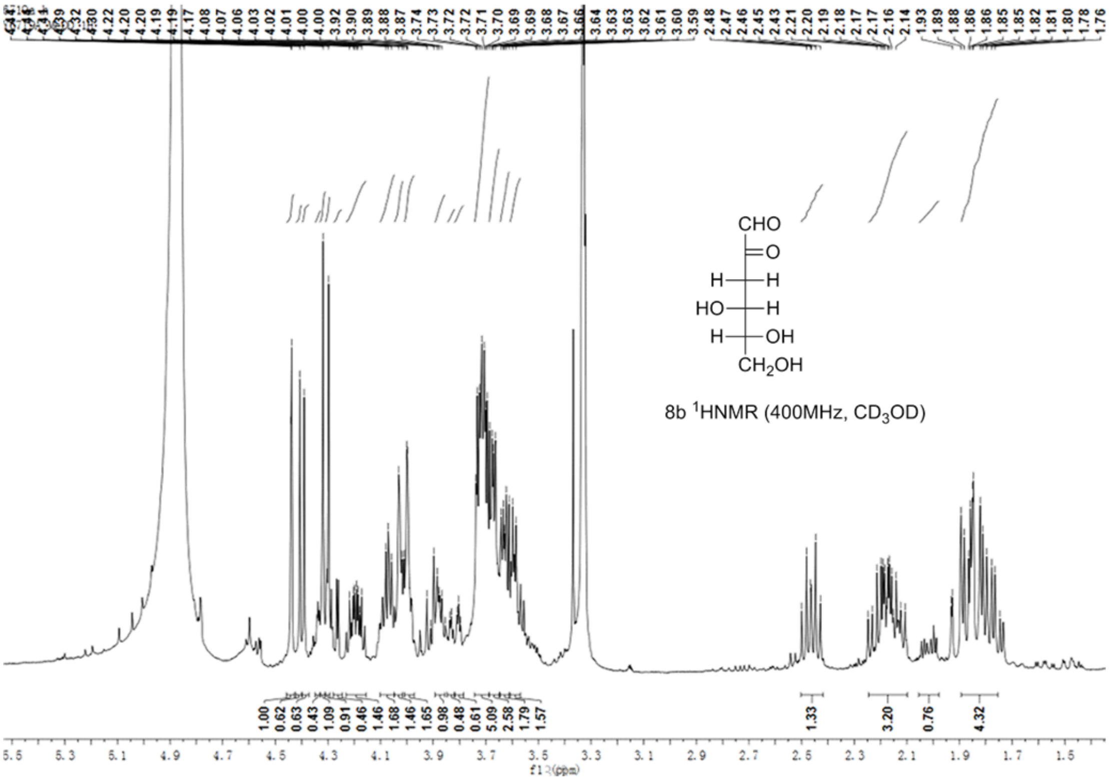
CHO
O
H
H
HO
H
H
OH
CH_2OH
8b ^{1}HNMR (400MHz, CD_3OD)
f1 (ppm)

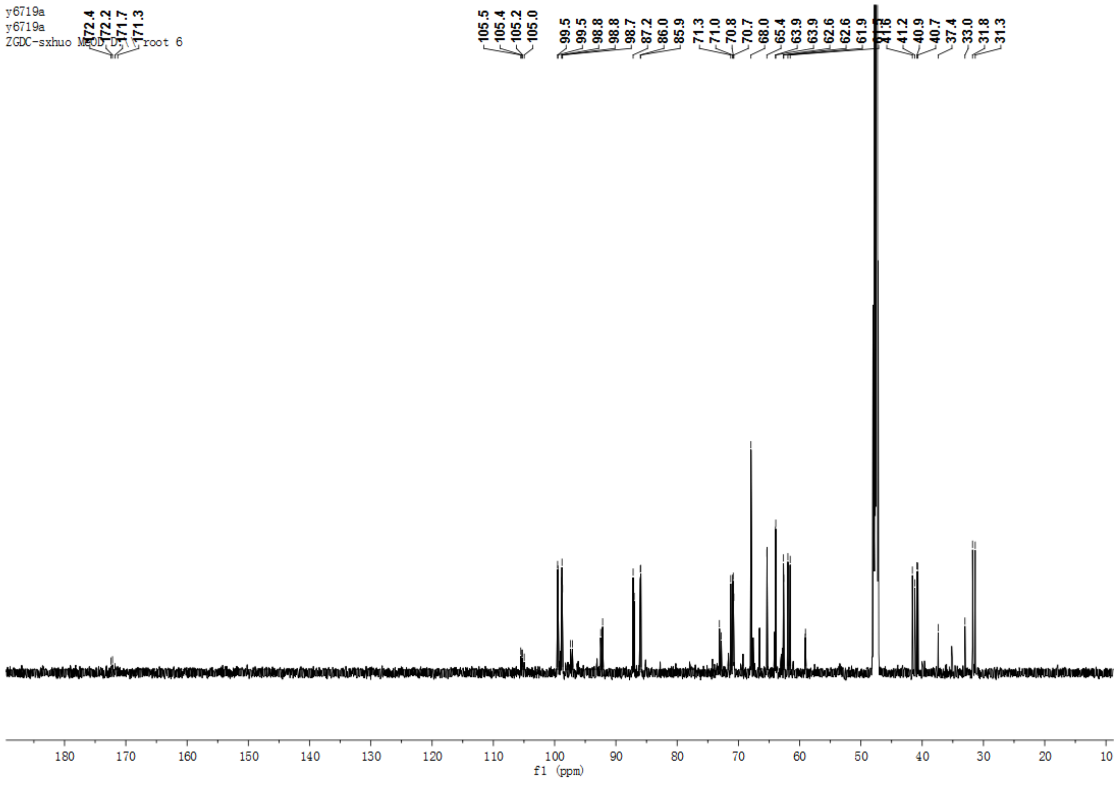
y6719a
y6719a
f1 (ppm)

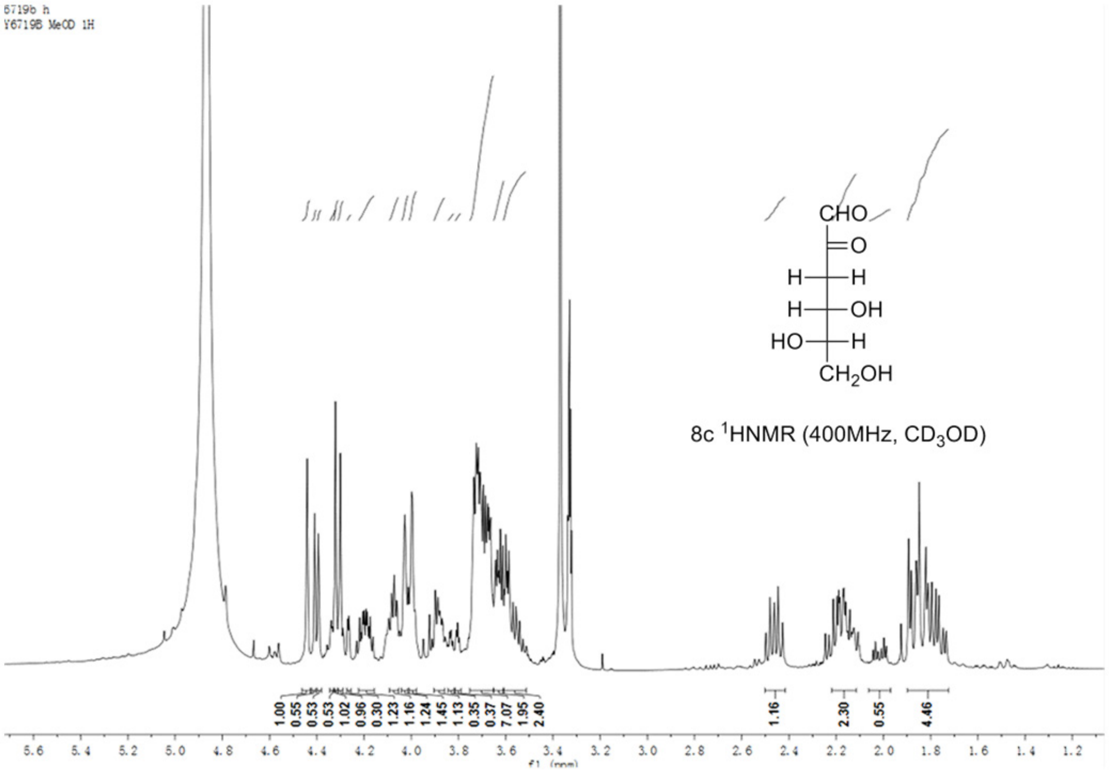

6719b h
Y6719B MeOD 1H
CHO
O
H
H
H
OH
HO
H
CH2OH
8c 1HNMR (400MHz, CD3OD)
f1 (ppm)

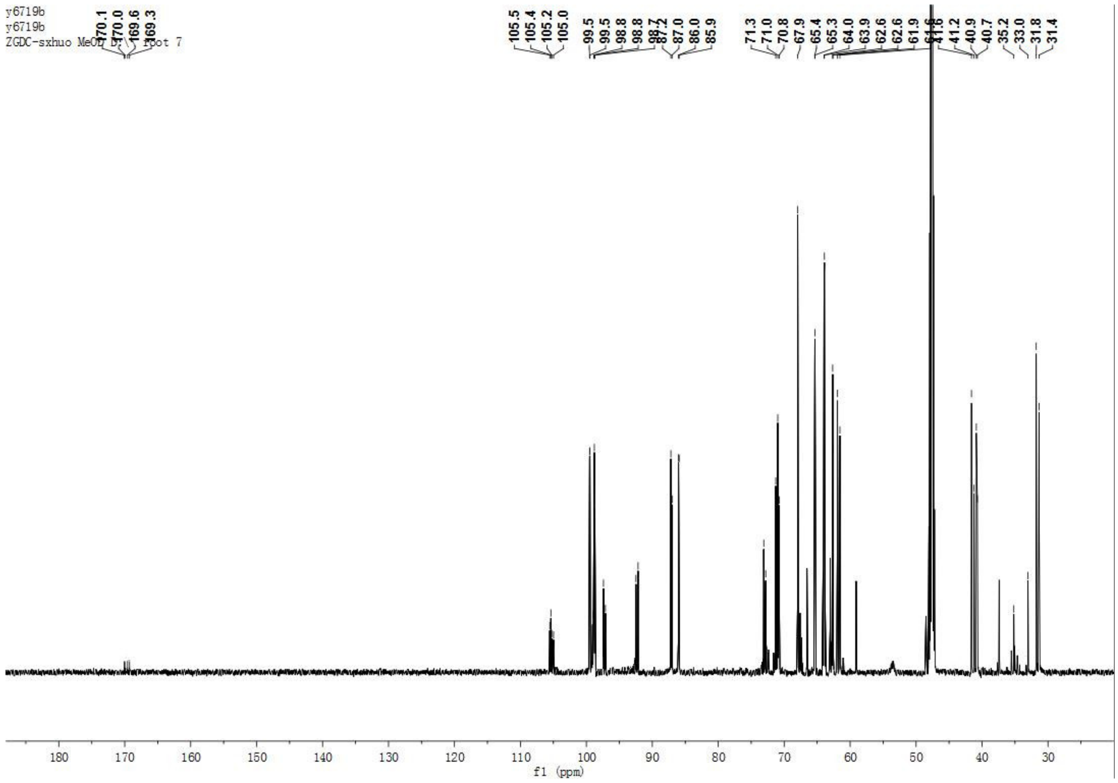

y6719b
y6719b
ZGDC-sxhuo MeOD
f1 (ppm)

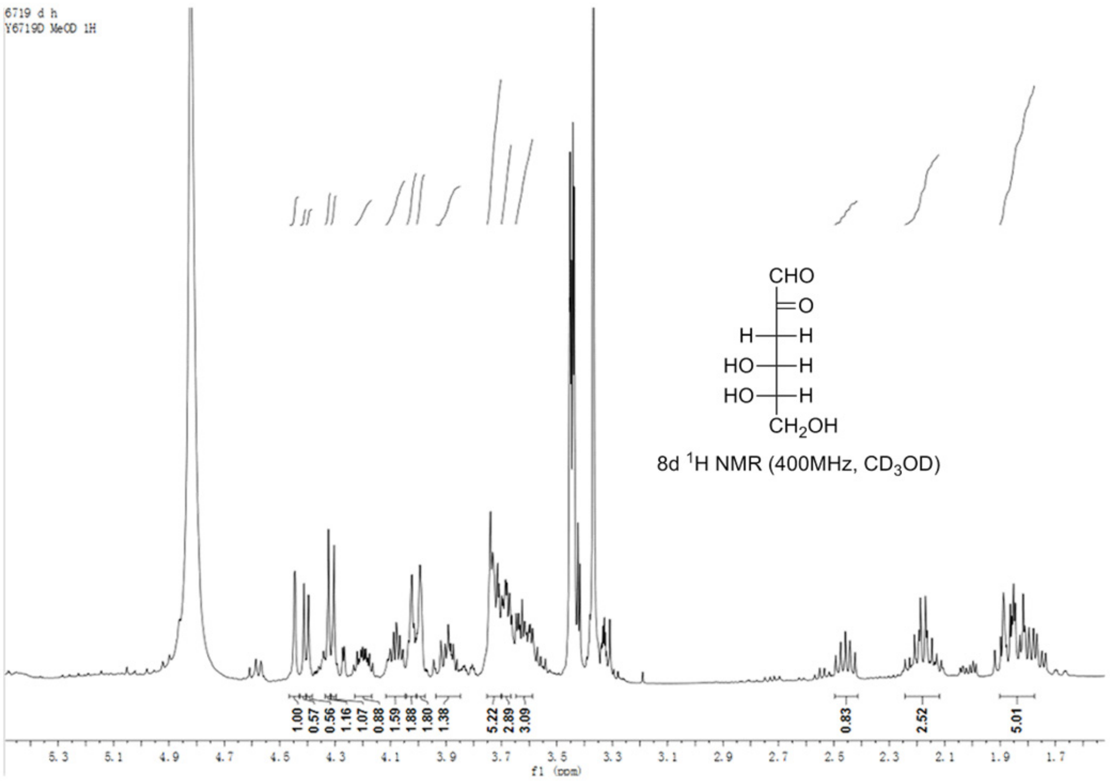
6719 d h
Y6719D MeOD 1H
CHO
=O
H—H
HO—H
HO—H
CH2OH
8d 1H NMR (400MHz, CD3OD)
1.00 0.57 0.56 1.16 1.07 0.88 1.59 1.88 1.80 1.38 5.22 2.89 3.09 0.83 2.52 5.01
f1 (ppm)

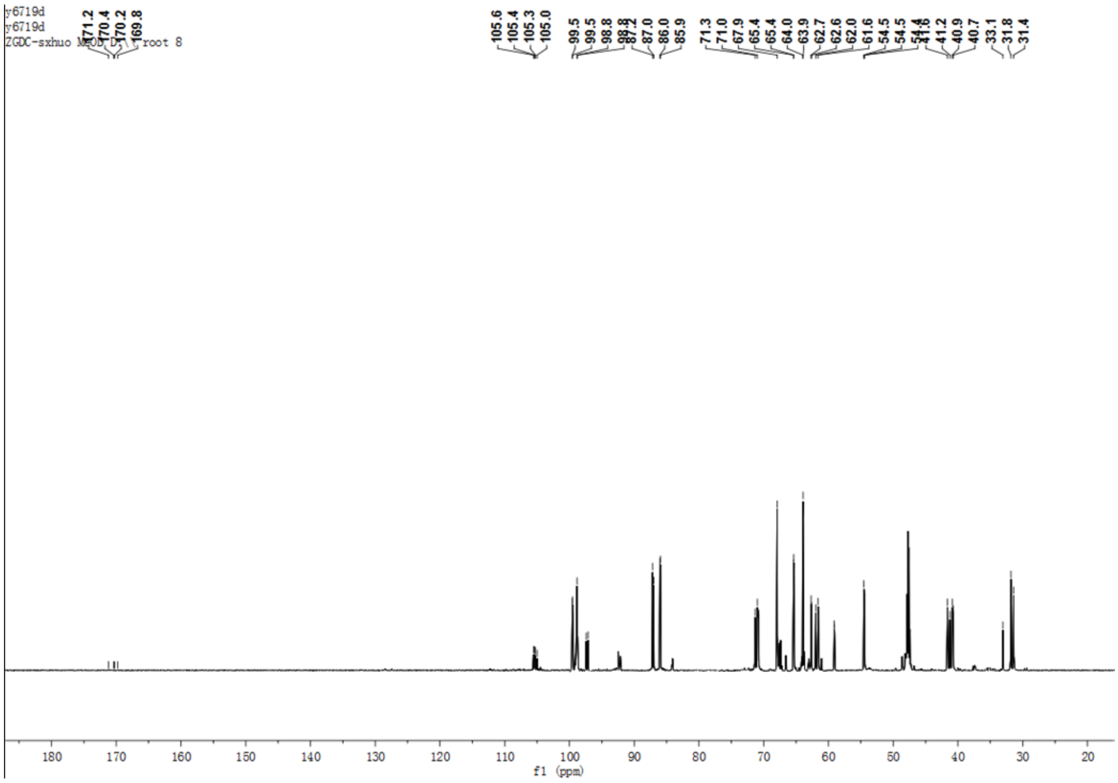
y6719d
y6719d
ZGDC-sxhuo MeOD D, root 8
171.2 170.4 170.2 169.8
105.6 105.4 105.3 105.0
99.5 99.5 98.8 98.8 87.2 87.0 86.0 85.9
71.3 71.0 67.9 65.4 65.4 64.0 63.9 62.7 62.6 62.0 61.6 54.5 54.5
41.2 40.9 40.7 33.1 31.8 31.4
f1 (ppm)

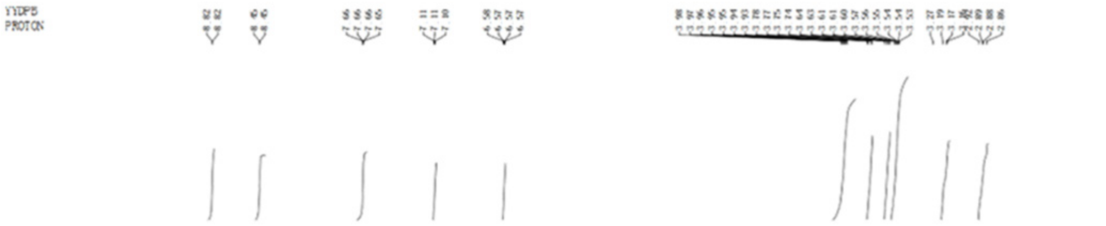

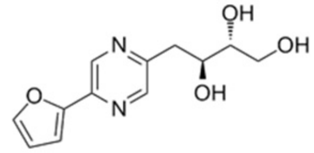

9a (2-(furan-2'-yl)-5-(2"*S*,3"*R*,4-trihydroxy-butyl)-1,4-diazine)

^{1}H NMR (400M, CD3OD)

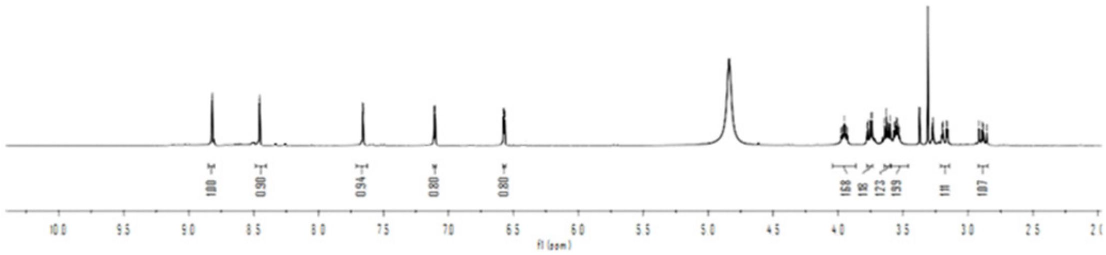

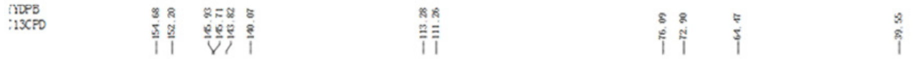

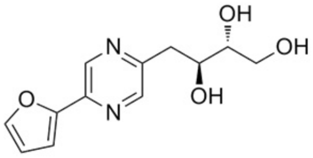

9a (2-(furan-2'-yl)-5-(2"S,3"R,4-trihydroxy-butyl)-1,4-diazine)

400M ^{13}C NMR

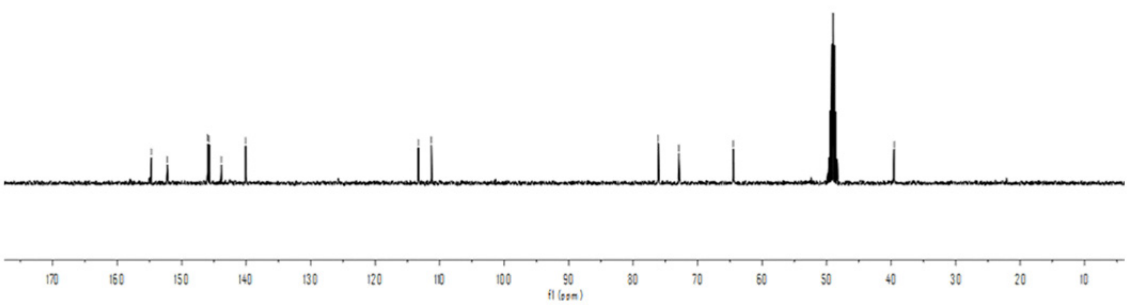

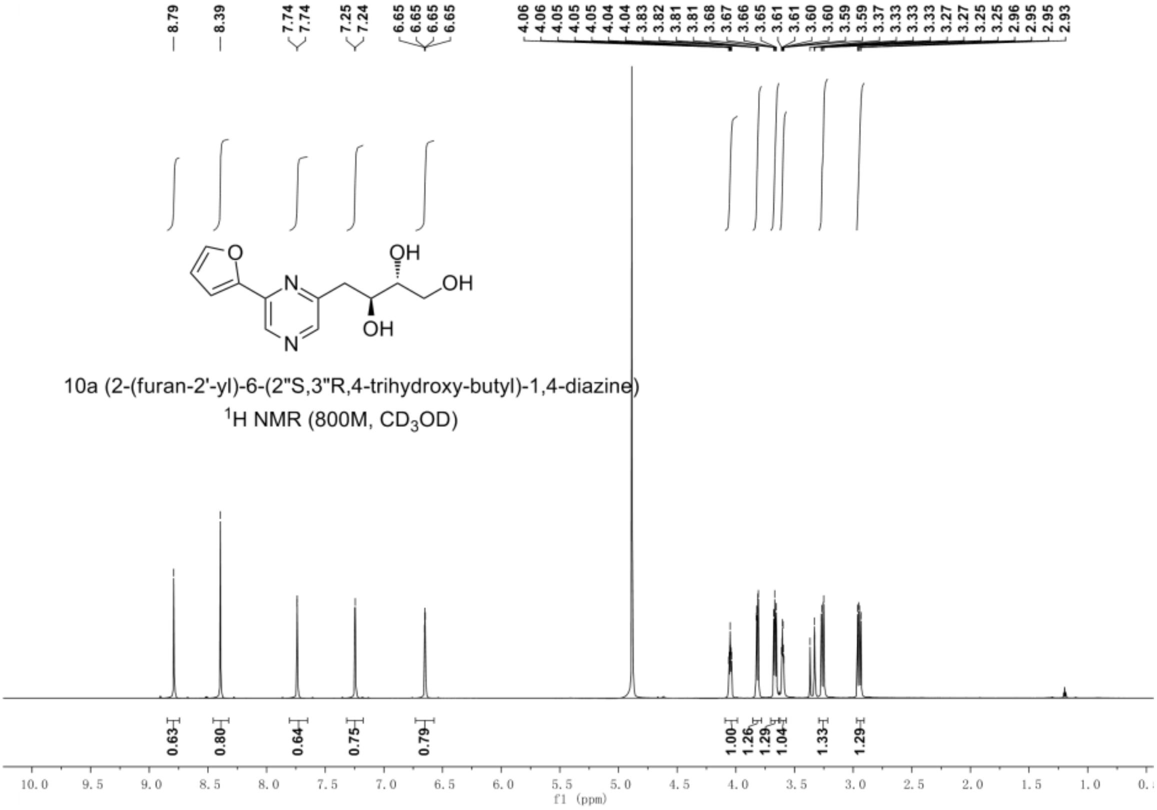
8.79
8.39
7.74
7.74
7.25
7.24
6.65
6.65
6.65
6.65
4.06
4.06
4.05
4.05
4.05
4.04
4.04
3.83
3.82
3.81
3.81
3.68
3.67
3.66
3.65
3.61
3.61
3.60
3.60
3.59
3.59
3.37
3.33
3.33
3.33
3.27
3.27
3.25
3.25
2.96
2.95
2.95
2.93
OH
OH
OH
O
N
N
10a (2-(furan-2'-yl)-6-(2"S,3"R,4-trihydroxy-butyl)-1,4-diazine)
^{1}H NMR (800M, CD_3OD)
0.63
0.80
0.64
0.75
0.79
1.00
1.26
1.29
1.04
1.33
1.29
f1 (ppm)

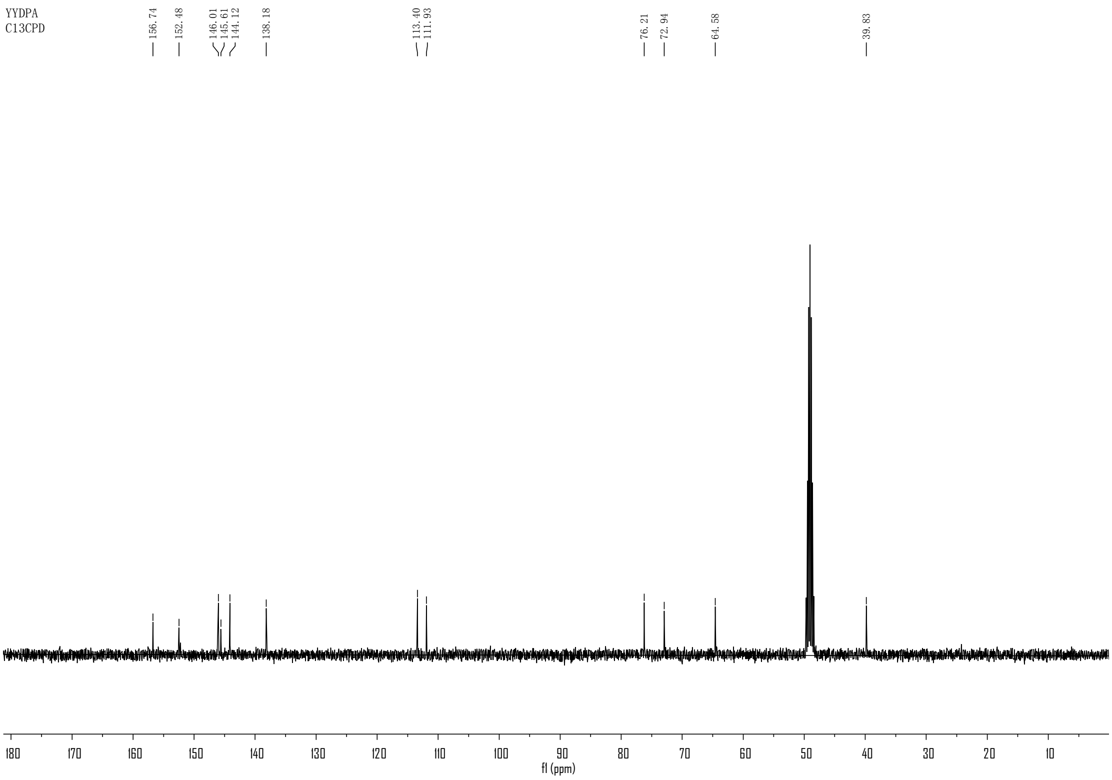
YYDPA
C13CPD
156.74
152.48
146.01
145.61
144.12
138.18
113.40
111.93
76.21
72.94
64.58
39.83
f1 (ppm)

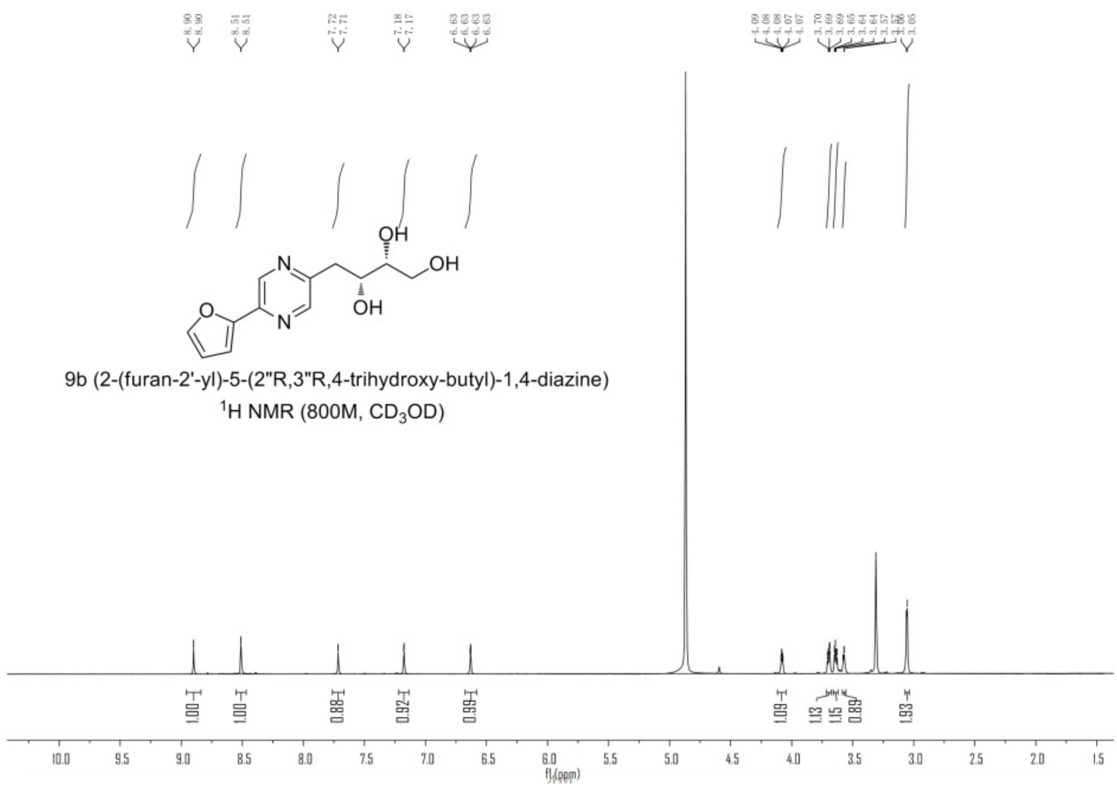
9b (2-(furan-2'-yl)-5-(2"R,3"R,4-trihydroxy-butyl)-1,4-diazine)
1H NMR (800M, CD3OD)
8.90 8.90 8.51 8.51 7.72 7.71 7.18 7.17 6.63 6.63 6.63 6.63
4.09 4.08 4.08 4.07 4.07 3.70 3.69 3.69 3.65 3.64 3.64 3.57 3.06 3.05
1.00 1.00 0.88 0.92 0.99 1.09 1.13 1.15 0.89 1.93
fl (ppm)

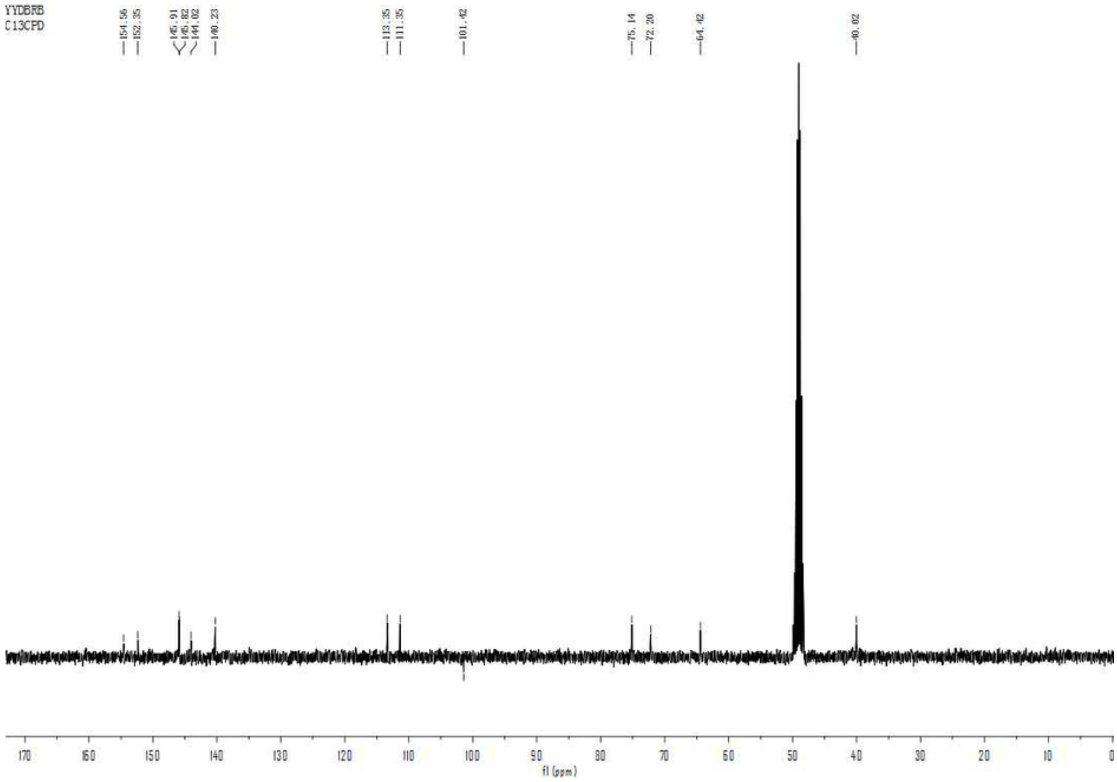
YYDBRB
C13CPD
154.56 152.35 145.91 145.82 144.02 140.23 113.35 111.35 101.42 75.14 72.20 64.42 40.02
fl (ppm)

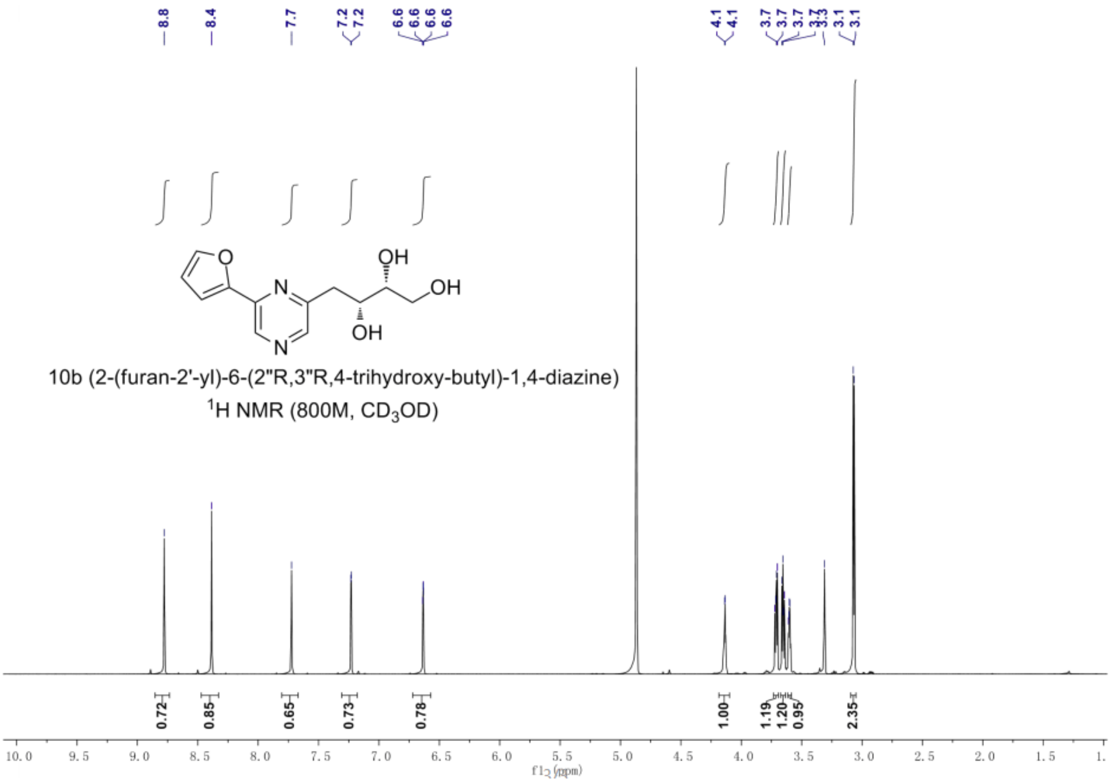
8.8
8.4
7.7
7.2
7.2
6.6
6.6
6.6
6.6
4.1
4.1
3.7
3.7
3.7
3.3
3.1
3.1
10b (2-(furan-2'-yl)-6-(2"R,3"R,4-trihydroxy-butyl)-1,4-diazine)
1H NMR (800M, CD3OD)
0.72
0.85
0.65
0.73
0.78
1.00
1.19
1.20
0.95
2.35
10.0 9.5 9.0 8.5 8.0 7.5 7.0 6.5 6.0 5.5 5.0 4.5 4.0 3.5 3.0 2.5 2.0 1.5
f1 (ppm)

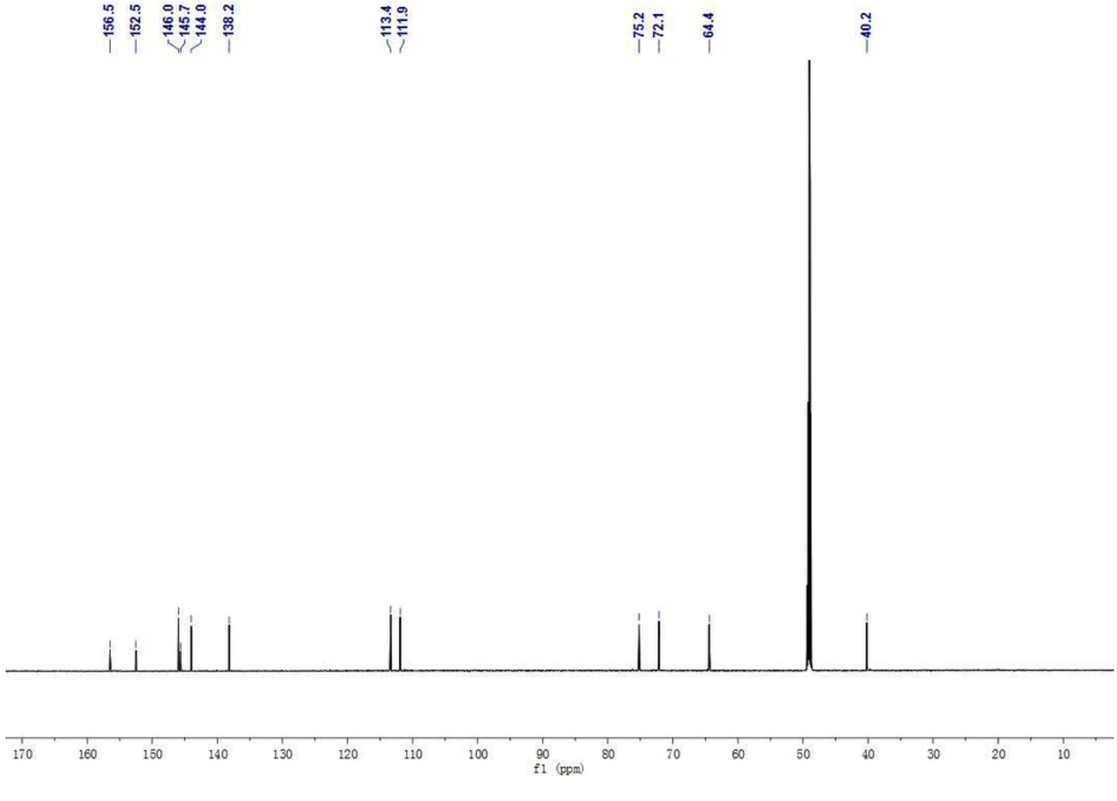
156.5
152.5
146.0
145.7
144.0
138.2
113.4
111.9
75.2
72.1
64.4
40.2
170 160 150 140 130 120 110 100 90 80 70 60 50 40 30 20 10
f1 (ppm)

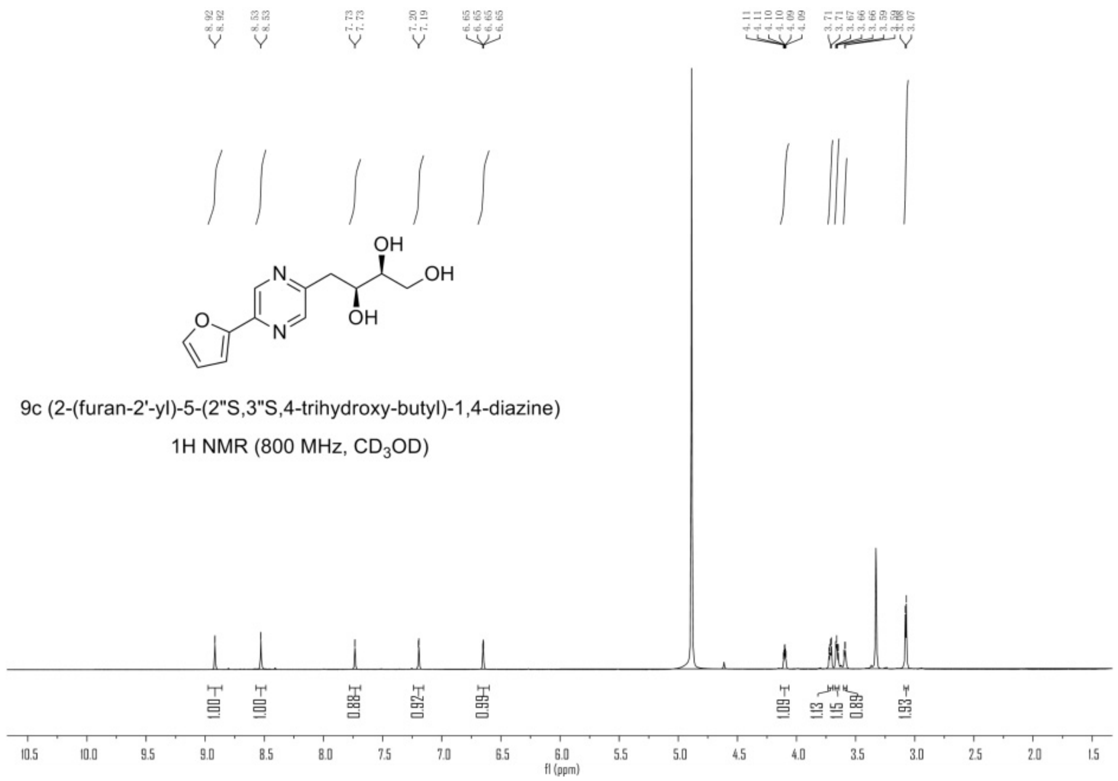
9c (2-(furan-2'-yl)-5-(2"S,3"S,4-trihydroxy-butyl)-1,4-diazine)
1H NMR (800 MHz, CD_3OD)
f1 (ppm)

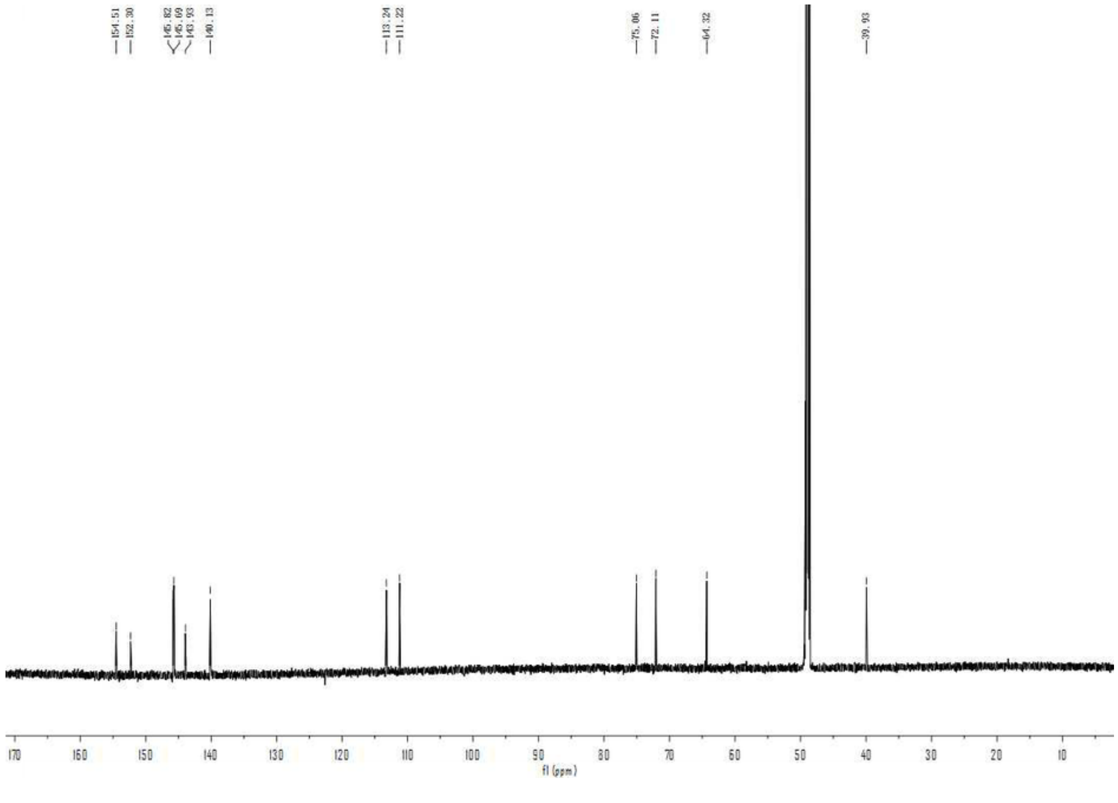
f1 (ppm)

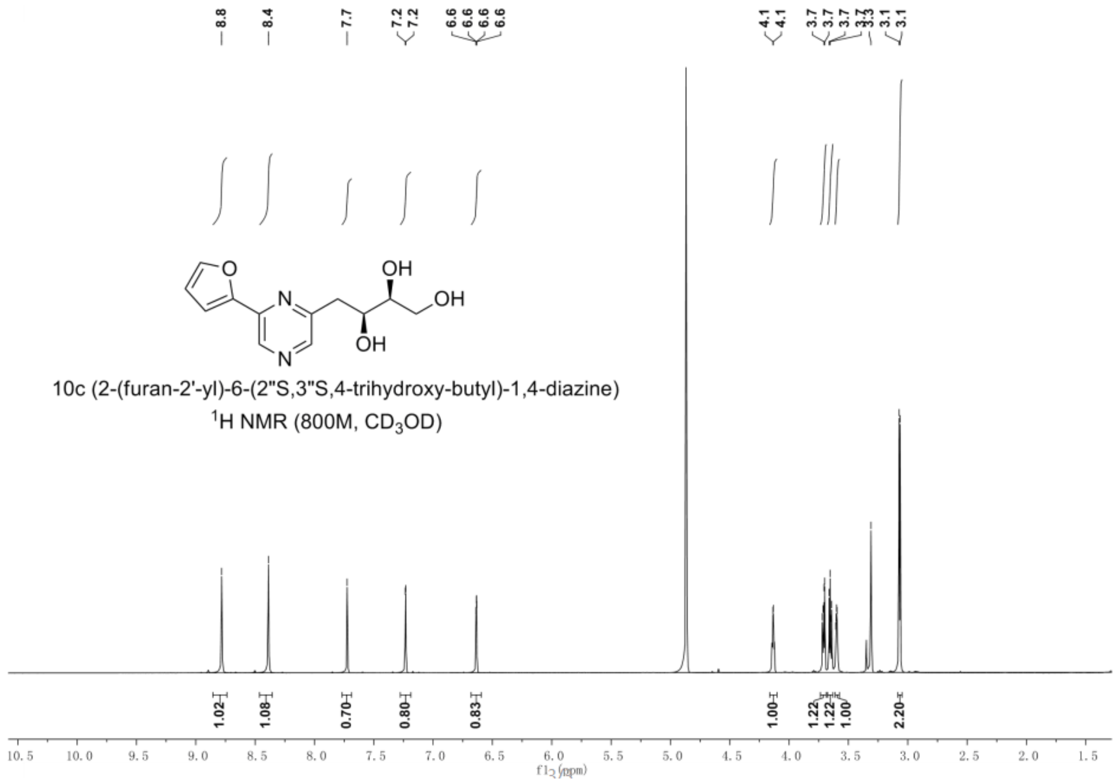
10c (2-(furan-2'-yl)-6-(2"S,3"S,4-trihydroxy-butyl)-1,4-diazine)
^{1}H NMR (800M, CD_3OD)
8.8
8.4
7.7
7.2
7.2
6.6
6.6
6.6
6.6
4.1
4.1
3.7
3.7
3.7
3.7
3.3
3.1
3.1
1.02
1.08
0.70
0.80
0.83
1.00
1.22
1.22
1.00
2.20
f1 (ppm)

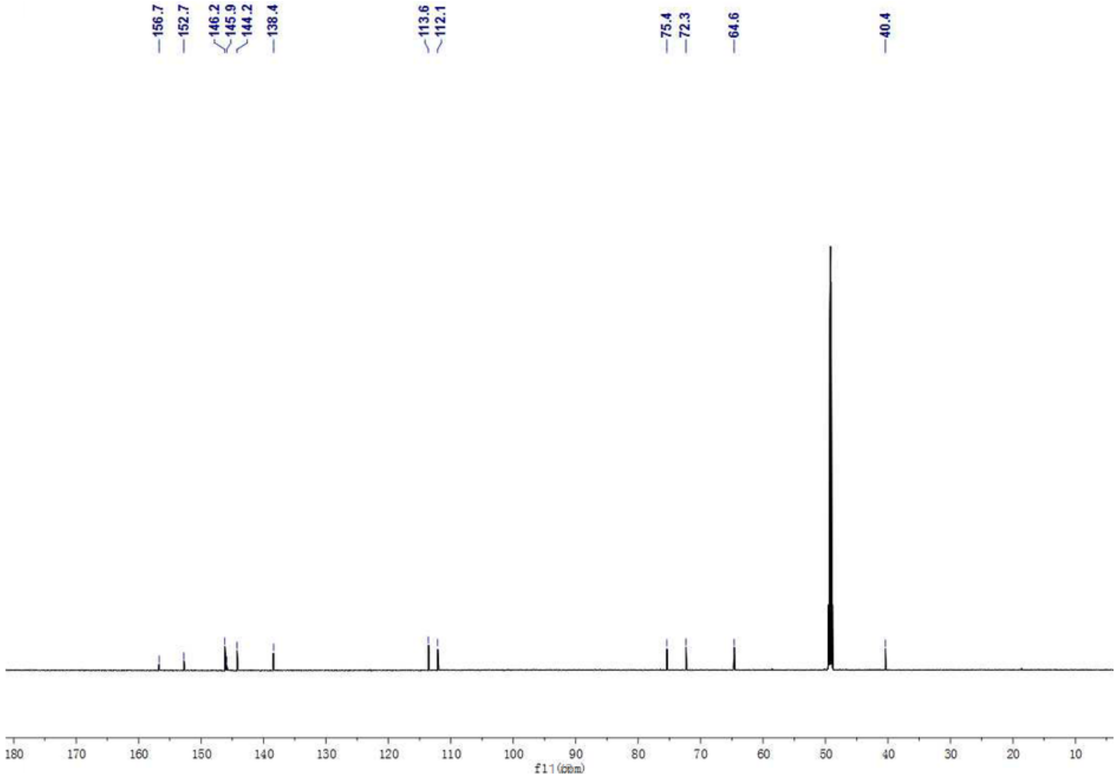
156.7
152.7
146.2
145.9
144.2
138.4
113.6
112.1
75.4
72.3
64.6
40.4
f1 (ppm)

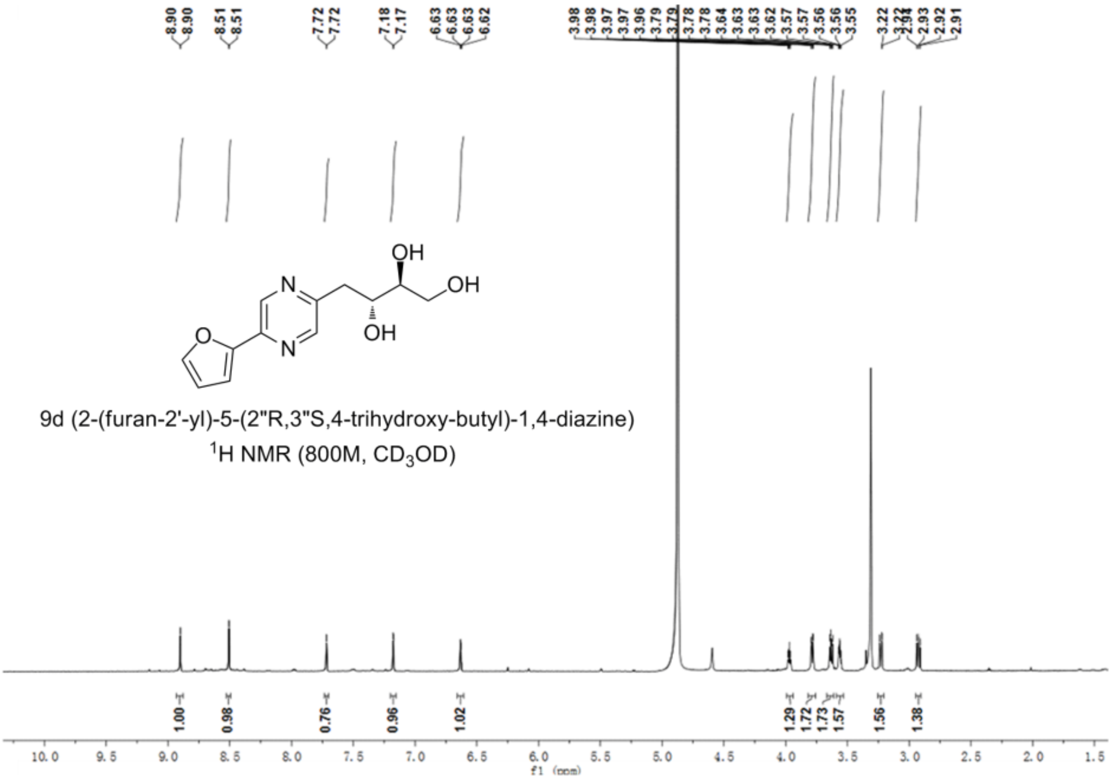
9d (2-(furan-2'-yl)-5-(2"R,3"S,4-trihydroxy-butyl)-1,4-diazine)
^{1}H NMR (800M, CD_3OD)
OH
OH
OH
N
N
O
1.00
0.98
0.76
0.96
1.02
1.29
1.72
1.73
1.57
1.56
1.38
f1 (ppm)

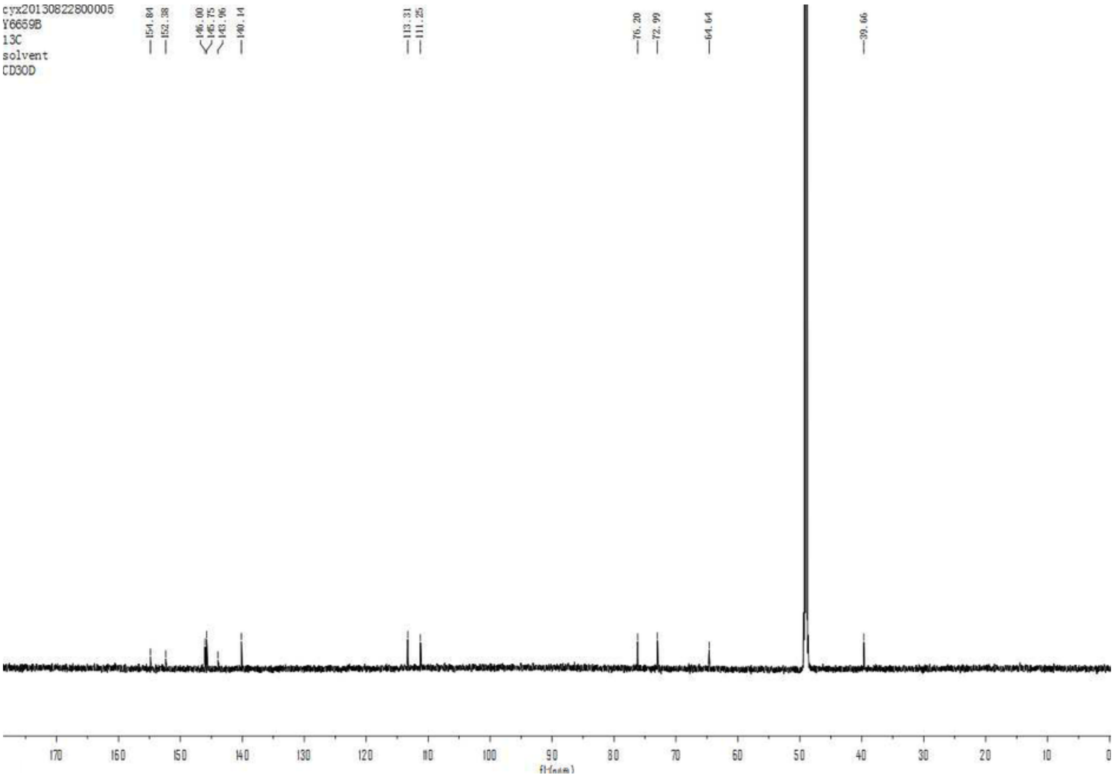
cyx20130822800005
Y6659B
13C
solvent
CD3OD
154.84
152.38
146.00
145.75
143.96
140.14
113.31
111.25
76.20
72.99
64.64
39.66
f1 (ppm)

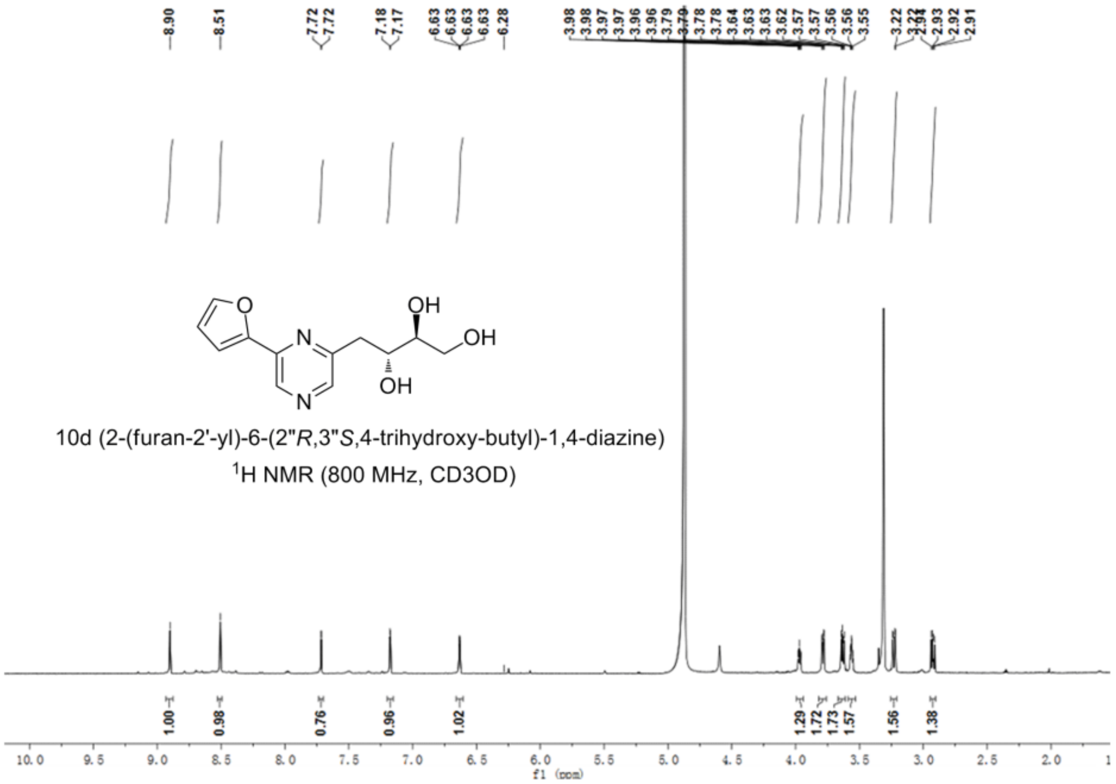

10d (2-(furan-2'-yl)-6-(2"*R*,3"*S*,4-trihydroxy-butyl)-1,4-diazine)

^{1}H NMR (800 MHz, CD3OD)

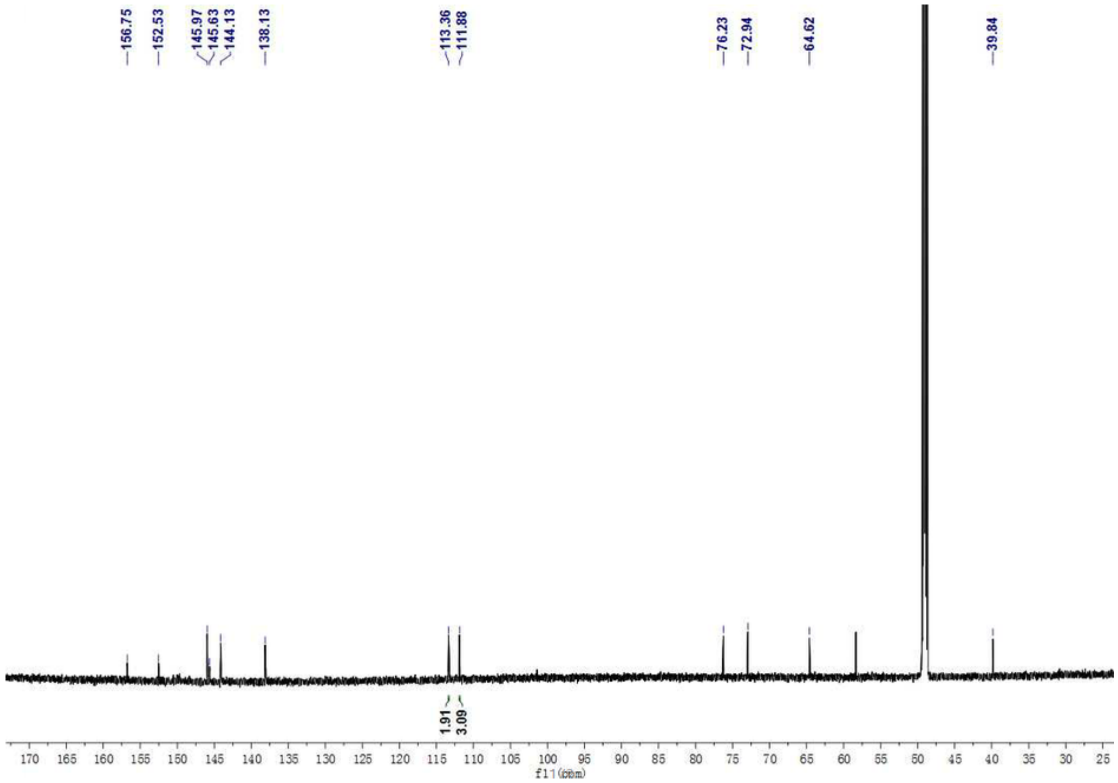

Supplement: Supplementary file 1 — Supplementary material, approximately 2.73 MB. [file 13659_2013_80_MOESM1_ESM.pdf]
